# A parallel methodology of adaptive Cartesian grid for compressible flow simulations

**DOI:** 10.1186/s42774-022-00108-y

**Published:** 2022-05-06

**Authors:** Xinyu Qi, Yuchen Yang, Linlin Tian, Zhenming Wang, Ning Zhao

**Affiliations:** 1 College of Aerospace Engineering, Nanjing University of Aeronautics and Astronautics, Nanjing, 210016 China; 2 State Key Laboratory of Mechanics and Control of Mechanical Structures, Nanjing University of Aeronautics and Astronautics, Nanjing, 210016 China

**Keywords:** Cartesian grids, AMR, Parallel computing, Compressible flow, Immersed boundary method

## Abstract

The combination of Cartesian grid and the adaptive mesh refinement (AMR) technology is an effective way to handle complex geometry and solve complex flow problems. Some high-efficiency Cartesian-based AMR libraries have been developed to handle dynamic changes of the grid in parallel but still can not meet the unique requirements of simulating flow around objects. In this paper, we propose an efficient Cartesian grid generation method and an information transmission approach for the wall boundary to parallelize the implementation of ghost-cell method (GCM). Also, the multi-valued ghost-cell method to handle multi-value points is improved to adapt to the parallel framework. Combining the mentioned methodologies with the open-source library p4est, an automatic and efficient simulation of compressible flow is achieved. The overall performance of the methodology is tested through a wide range of inviscid/viscous flow cases. The results indicate that the capability and parallel scalability of the present numerical methodology for solving multiple types of flows, involving shock and vortices, multi-body flow and unsteady flows are agreeable as compared with related reference data.

## Introduction

Concerns about the cost and reliability of grid generation were raised repeatedly in the surveys and workshops of the computational fluid dynamics (CFD) community [1]. Compared with structured/unstructured grids, Cartesian grids have several advantages in automatic grid generation, high mesh quality, and easy coupling with high-order schemes, especially in applications that can benefit from the AMR method [2]. Therefore, the simulation of incompressible/compressible flow based on Cartesian grids has always been one of the CFD research topics.

However, in the Cartesian grid system, the grids can not completely coincide with the boundaries of the object surface. Thus the resulting wall boundaries are expressed as a set of staircase-like facets [3]. Accordingly, additional treatments for the wall boundaries are required to recover smooth surface geometries. Generally, there are mainly two types of existing methods to reproduce smooth wall boundaries on a non-conforming Cartesian grid: the cut-cell methods [4], and the immersed boundary method (IBM) [5, 6]. The cut-cell method has the advantages of generating body-fitted grids automatically and ensuring conservation at the boundary, but the intersection calculation at the wall boundary is complex and special treatments are required due to the extremely small cells generated by cell reshaping. Alternatively, by adding a body forcefield in the momentum equation to simulate the presence of the immersed boundaries, IBM can avoid complex geometrical algorithms, thereby maintaining simplicity and robustness of grid generation, especially for the simulation of flows with complex and/or moving boundaries. Nowadays, IBM has been widely used in both inviscid and viscous flow [7, 8]. Tamaki et al. [9] and Constant et al. [10] applied the IBM method in high Reynolds number flow simulations and made significant progress. Based on IBM, the ghost-cell method (GCM) was first proposed by Forrer et al. [11] and applied to the problem of inviscid compressible flow, and then Dadone et al. [12] conducted in-depth and detailed research on the ghost-cell method. The main idea of GCM is to assume that the object is embedded in the flow field, and the boundary condition is imposed on the ghost-cell inside the object through reconstructions. Regarding the latest development and application of IBM, interested readers are encouraged to refer to [3] and the references therein.

On the other hand, AMR is used in the Cartesian grid system to play its advantages fully. Because it can solve problems dealing with phenomena appearing at multiple and different spatial and temporal scales, it has succeeded in multiphase flow [13], flow with complex geometry [14], turbulent flow [15], etc. Generally, the cell-based AMR method employs tree structures to store the mesh. On the basis of this method, the specific cell can be easily refined and coarsened by recursively dividing them into sub-cells at a fixed scale. However, the adaptive Cartesian grid can not be properly extended in parallel computer architectures due to the particularity of the storage data structure, grid traversal algorithm, and neighbor recursive search algorithm. The appearance of applications with new AMR frameworks such as CHOMBO [16] or p4est [17, 18] gives us the possibility to solve the issue. Recently, p4est has attracted wide attention because of the benefit of not having strict modularity restrictions. It was successfully applied in the refinement of CAD surfaces [19], flow simulation of hexahedron body-fitted adaptive grid [20], and is also used in a new version of the noted finite element library deal.II [21].

However, we noticed that most researches mentioned above are simple flow without objects. When employing these AMR frameworks to deal with compressible flows containing objects, it will bring some new difficulties. These difficulties mainly come from the parallelization of GCM, which are usually not considered in the above AMR framework. First of all, the above libraries are not specifically designed for the flow around objects, while it is necessary for GCM to determine the cells that intersect with the object surface when generating the grid. Secondly, the MPI communication of some special cells also needs to be re-planned. Some ghost-cells and their reference points might be in the different processes, while the ghost layer provided for communication in these AMR frameworks might not be enough for GCM. In addition, the multi-valued ghost-cell method [22] generally used to handle thin objects also brings challenges when parallelized. Storing all possible multi-value points for ghost-cell will impose a huge burden on the parallel framework and communications, while ignoring the multi-value points will make the flow simulation of delta wings and other shapes unavoidable errors. In response to the above- mentioned problems, the main contributions of this paper are specifically manifested in three aspects. First we built a module for grid generation and used a fast intersection algorithm based on the axis aligned bounding box (AABB) theorem [23] to classify cells. The mesh generator can generate adaptive grids with 15 million cells in parallel for complex 3D geometry (such as DLR-F6) in 10 minutes on a server with two Intel(R) Xeon(R) E5-2680 V3 CPUs (48 cores). Second, special communication relationships are established for all the ghost-cells and their corresponding points that might be in different processes to achieve simple and efficient parallel communication. The third aspect is that the algorithm for finding multi-value points is improved and combined with the communication relationship mentioned above to make it more suitable for complex three-dimensional shapes under the parallel framework. Eventually, by combining the in-house GCM-based automatic serial flow solver with p4est library, and then integrating the above methods, a new parallel adaptive Cartesian solver was developed, which is named CABA (CArtesian Body-fitting Adaptive). It can automatically generate computational grids for arbitrary three-dimensional objects and can solve multi-body and unsteady problems of inviscid and laminar flow with ghost-cell method for the wall boundary condition.

This paper is organized as follows. In Sec. 2, the relating methodologies, including the numerical approach, mesh generation, ghost-cell method and parallelization method, are presented. Numerical results, including parallel tests and inviscid/viscous flow tests, are discussed in Sec. 3 to validate the capability of CABA. Finally, conclusions are drawn in Sec. 4.

## Numerical methodology

### Governing equations and numerical approach

The compressible Navier-Stokes equations in the integral and conservation form are considered, which can be written as follows:

1 $$ \frac{\partial}{\partial{t}} \int_{\Omega} \mathbf{W}d\Omega + \oint_{\partial{\Omega}} (\mathbf{F}_{c} - \mathbf{F}_{v}) d\mathbf{S}=0,  $$

where *∂Ω* is the boundary of the control volume, **W** is the vector of conserved variable, **F**_*c*_ and **F**_*v*_ correspond to the vectors of the inviscid and viscous flux respectively. The vectors are given as:

2 $$ \begin{aligned} \mathbf{W}=\left[\begin{array}{c}\rho\\\rho{u}\\\rho{v}\\\rho{w}\\\rho{E} \end{array}\right], \mathbf{F}_{c}=\left[\begin{array}{c}\rho{V}\\\rho{u}{V}+n_{x}{P}\\\rho{v}{V}+n_{y}{P}\\\rho{w}{V}+n_{z}{P}\\\rho{H}{E} \end{array}\right], \mathbf{F}_{v}=\left[\begin{array}{c}{0}\\n_{x}{\tau_{{x}{x}}} + n_{y}{\tau_{{x}{y}}} + n_{z}{\tau_{{x}{z}}}\\ n_{x}{\tau_{{y}{x}}} + n_{y}{\tau_{{y}{y}}} + n_{z}{\tau_{{y}{z}}}\\ n_{x}{\tau_{{z}{x}}} + n_{y}{\tau_{{z}{y}}} + n_{z}{\tau_{{z}{z}}}\\ n_{x}{\Theta_{x}} + n_{y}{\Theta_{y}} + n_{z}{\Theta_{z}} \end{array}\right], \end{aligned}  $$

where *ρ* is the density; *u*,*v*,*w* are the velocity components in *x*,*y*,*z* directions, respectively; *P* is the pressure; *E* and *H* are the total energy and the total enthalpy per unit mass. *τ*_*ij*_ are the viscous stress tensor for Newtonian fluids, which are defined as:

3 $$ \begin{aligned} &\tau_{{x}{x}}=\lambda{(\frac{\partial{u}}{\partial{x}} + \frac{\partial{v}}{\partial{y}} + \frac{\partial{w}}{\partial{z}}) + 2{\mu}\frac{\partial{u}}{\partial{x}}},\\ &\tau_{{y}{y}}=\lambda{(\frac{\partial{u}}{\partial{x}} + \frac{\partial{v}}{\partial{y}} + \frac{\partial{w}}{\partial{z}}) + 2{\mu}\frac{\partial{v}}{\partial{y}}},\\ &\tau_{{z}{z}}=\lambda{(\frac{\partial{u}}{\partial{x}} + \frac{\partial{v}}{\partial{y}} + \frac{\partial{w}}{\partial{z}}) + 2{\mu}\frac{\partial{w}}{\partial{z}}},\\ &\tau_{{x}{y}}=\tau_{{y}{x}}=\mu(\frac{\partial{u}}{\partial{y}} + {\partial{v}}{\partial{x}}),\\ &\tau_{{x}{z}}=\tau_{{z}{x}}=\mu(\frac{\partial{u}}{\partial{z}} + {\partial{w}}{\partial{x}}),\\ &\tau_{{y}{z}}=\tau_{{z}{y}}=\mu(\frac{\partial{v}}{\partial{z}} + {\partial{w}}{\partial{y}}), \end{aligned}  $$

where *μ* is molecular viscosity coefficient calculated by the Sutherland law [24], and *λ*=−2/3*μ* with Stokes hypothesis.

**Fig. 1:**
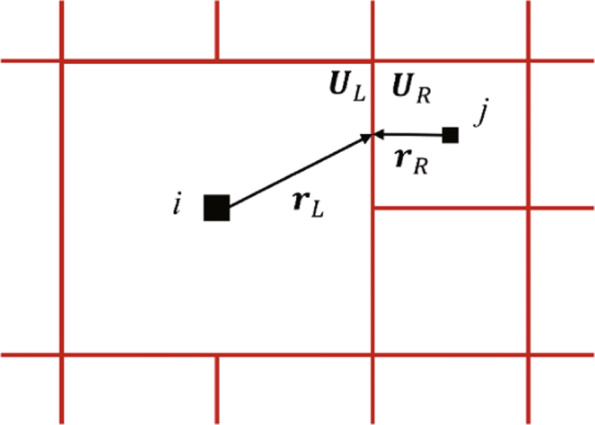
Reconstructions of the states on adaptive Cartesian grid

For the compressible Euler/Navier-Stokes equations, the flow states need to be reconstructed on the left and right sides of an interface of neighboring control volumes, as sketched in Fig. 1. The governing equations are discretized using the finite volume formulation, and a cell-centered, second-order method is used in this paper [24]:

4 $$ \mathbf{U}_{L}=\mathbf{U}_{i} + \mathbf{\Phi}_{i}\cdot(\nabla \mathbf{U}_{i}\cdot \mathbf{r}_{L}), \mathbf{U}_{R}=\mathbf{U}_{j} + \mathbf{\Phi}_{j}\cdot(\nabla \mathbf{U}_{j}\cdot \mathbf{r}_{R}),  $$

where **r**_*L*_ and **r**_*R*_ represent the vector from the left and right cell center to the face midpoint. **U**_*i*_ is the gradient at cell center *i*, **U**_*j*_ is the gradient at cell center *j*, and they are all calculated by the Green-Guass method [25] in this paper. **Φ**_*i*_ is the limiter for cell center *i*, **Φ**_*j*_ is the limiter for cell center *j*, and they are all calculated by the Venkatakrishnan limiter [26]. The inviscid flux **F**_*c*_ at each cell interface is computed by the HLLC scheme developed by Toro et al. [27], and the viscous flux **F**_*v*_ is approximated by using 2nd order accurate central difference scheme in Ref. [28]. The solution is updated by using the explicit three-stage third-order Runge-Kutta method [24], and the CFL number is set to 0.8 for all examples in this paper.

### Mesh generation

**Fig. 2:**
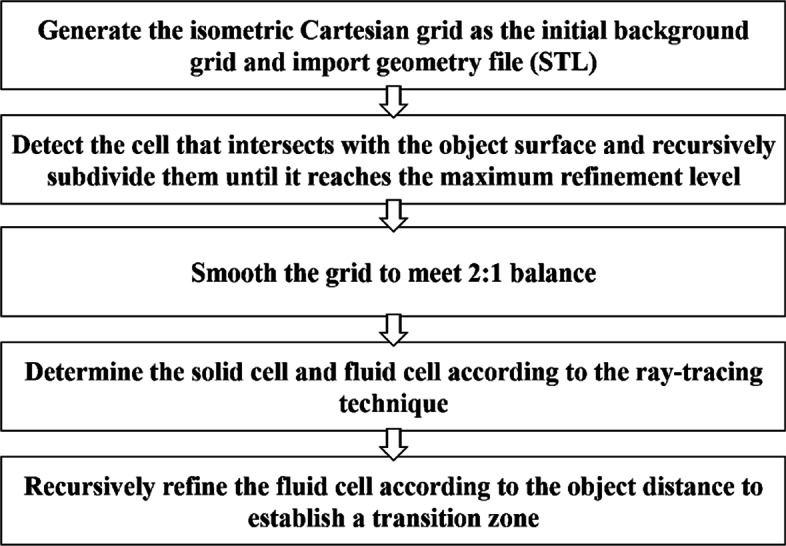
The overall framework of the grid generation process in CABA

**Fig. 3:**
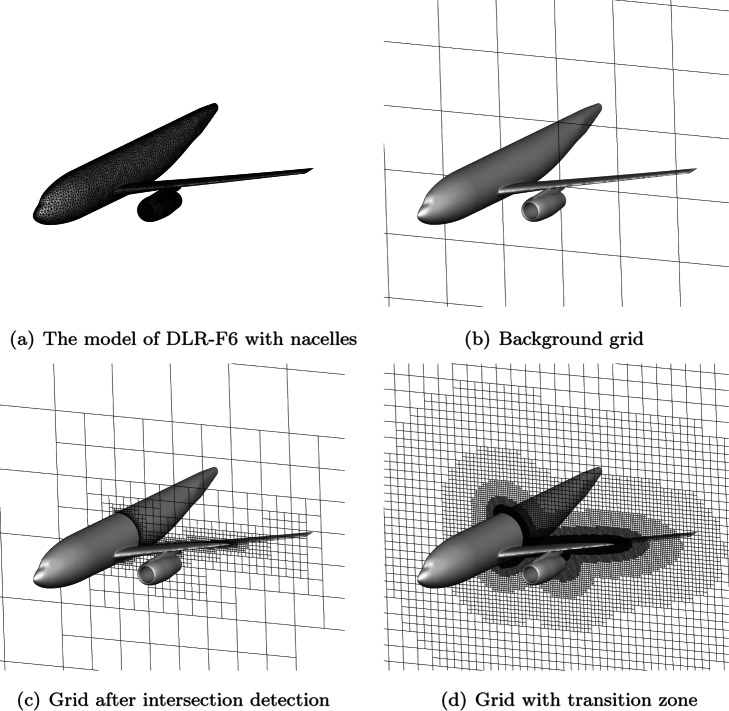
The changes of the grid during the generation process of DLR-F6 model

The process for generating an adaptive Cartesian grid is shown in Fig. 2. The entire process is highly parallelized and automated. All the user needs to do is to specify the input geometry file, the calculation domain and the maximum level of refinement. Figure 3 shows the changes of the grid during the generation process of DLR-F6 model. In addition, it should be emphasized that the second and fifth steps of the process are critical to the efficiency of generating grid and the quality of the grid. Therefore, the detailed strategies for these two steps are given below.

The second step in the process is to determine the intersection of the cell and the object surface. For complex three-dimensional geometric shapes, the number of a high-quality Cartesian grid often reaches to the order of tens of millions. For such a situation, an efficient algorithm for judging the intersection of the object surface and the cell is highly needed. Here, a fast intersection test [23] that is based on the axis aligned bounding box (AABB) theorem is employed in our work. It is based on Plucker coordinate and tests the ray against the silhouette of the AABB, instead of testing against individual faces of the box or comparing intersection intervals. The algorithm is performed using only dot products and comparisons while the classic algorithm requires division. Its computational simplicity results in excellent performance. After quickly identifying the cells that intersect the wall boundary, these cells will be refined recursively until the maximum refinement level is reached as shown in Fig. 3(c).

The fifth step in Fig. 2 is to establish the transition zone of the grid. Refining only the intersecting cells may result in the cell size in the boundary layer being too large, thus the obtained mesh needs to be further refined. The precise distance to the object surface is calculated for the cells with level *R* and *R*−1, where *R* is the maximum level of refinement. The rest of the cells only calculate the rough distance to the object surface to save calculations. Then the cells will be recursively refined if the distance satisfies the following relationship:

5 $$ D< r\cdot h/2,  $$

where *D* is the distance to the object surface, *r* is the level of the cell and *h* is the length of the cell. Then, the resulting grid fits the object surface model to a large extent, and the size of the grid is guaranteed in the boundary layer and nearby areas. Figure 3(d) shows the grid with transition zone.

According to our experience, the maximum refinement level and the number of surface meshes of the object are the key factors that determine the time to mesh generation. In order to capture the main flow phenomenon in specific flow, the minimum size of the grid should be estimated in advance, so that the maximum refinement level is also determined. The minimum size of the triangular mesh of the surface should preferably be limited to match the level, so that the time to generate the mesh can be minimized.

**Fig. 4:**
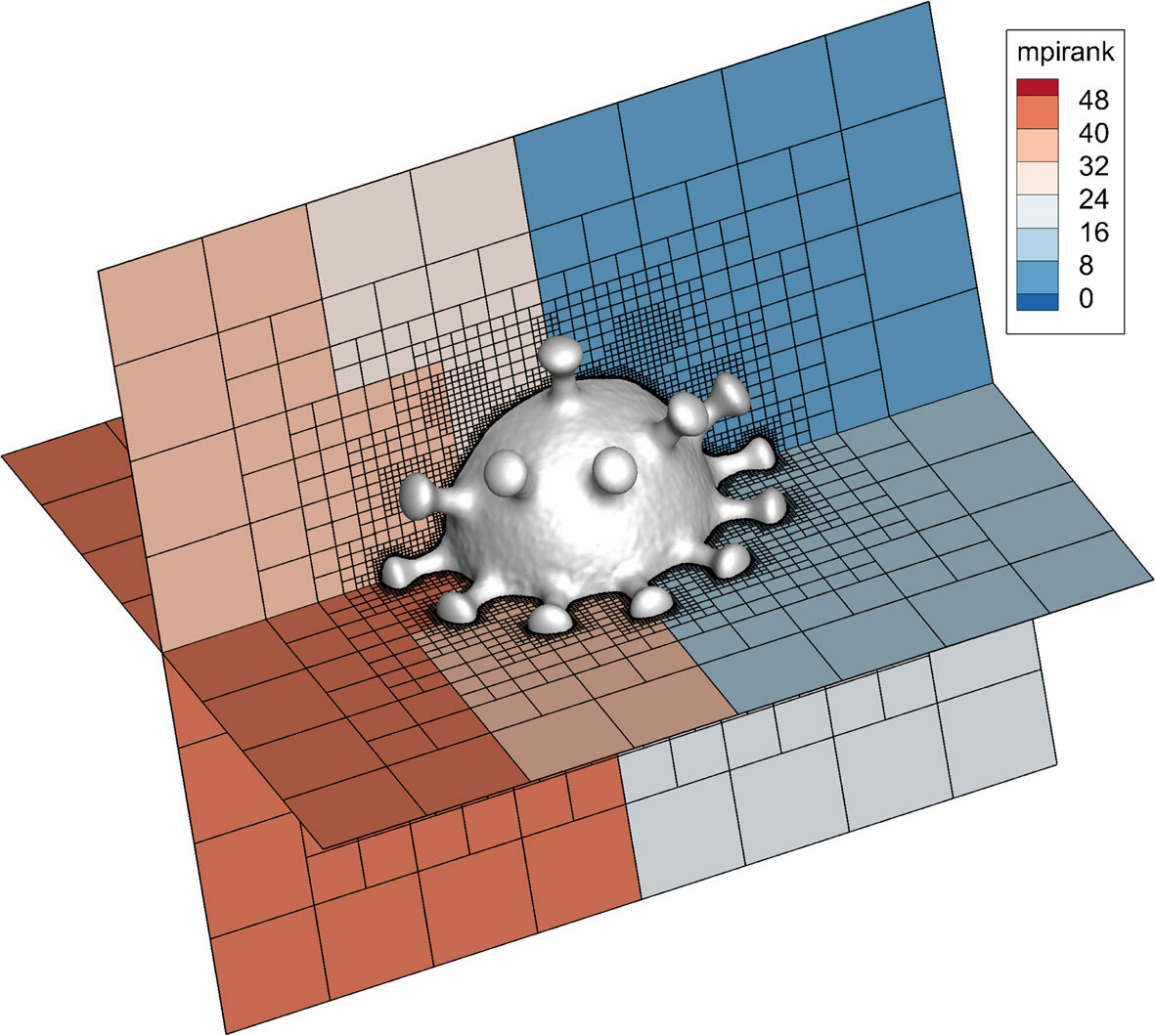
The adaptive grid generated by CABA for COVID-19 virus

After the entire process, a high-quality computational grid is obtained. For the model of DLR-F6, it contains 16,280 triangular surface meshes and it takes about 600 seconds to generate the final grid of 15 million. For models with more complex surface, such as the COVID-19 model with 188,280 triangular surface meshes, the time to generate 3 million grids is about 500 seconds. The final adaptive grid is shown in Fig. 4. Furthermore, for arbitrary shapes, CABA can automatically and efficiently generate high-quality computational grids without any manual intervention. This is an important part of solving large-scale and complex problems on the Cartesian grid. The cases were tested on a server with two Intel(R) Xeon(R) E5-2680 V3 CPUs (48 cores).

After the above process, high-quality grids can be obtained. But they are not capable of simulating complex flows. In order to accurately capture flow phenomena such as shock waves and vortices, we perform mesh refinements based on the characteristic of the flow field. The following criteria are mainly used in this paper to capture the special flow field structures: the divergence of velocity, the curl of velocity, or both of them. Their specific expressions are as follows [29]:

6 $$ \tau_{ci}=|\nabla \times V|h_{i}^{\frac{r+1}{r}}, \tau_{di}=|\nabla \cdot V|h_{i}^{\frac{r+1}{r}}, \sigma_{c}=\sqrt{\frac{\sum_{i=1}^{N}{\tau_{ci}^{2}}}{N}}, \sigma_{d}=\sqrt{\frac{\sum_{i=1}^{N}{\tau_{di}^{2}}}{N}},  $$

where *N* is the total number of cells and *h*_*i*_ is the length scale of the cell, computed as $h_{i}=\sqrt [r]{\Omega _{i}}$ with *Ω* being the volume of the cell. Here we use the standard deviations of divergence and curl as the sensors, the conditions can be described as:

1. refine: when *τ*_*ci*_>*w*_1_*σ*_*c*_ or *τ*_*di*_>*w*_2_*σ*_*d*_,
2. coarsen: when *τ*_*ci*_<*w*_3_*σ*_*c*_ and *τ*_*di*_<*w*_4_*σ*_*d*_,

where *w*_*i*_(*i*=1,2,3,4) are adjustable coefficients based on different problems.

After two kinds of adaptations, large-scale and high-quality meshes for arbitrary complex shapes are generated, besides, through mesh refinements the steady and unsteady flow phenomena can be automatically captured. Note that the whole process is automatic and efficient without manual intervention.

### Parallel computing of adaptive Cartesian grid

Since the adaptive Cartesian grid is continuously refined and coarsened with the flow characteristics, it brings many difficulties in large-scale high-performance computing, such as load balancing, reducing communication cost, and search algorithm. And, generally, the storage of tree structure used in AMR is made in linear arrays to increase efficiency. However, this method causes a bad cache locality making it difficult to parallelize. Up to now, there are many cell-based parallel AMR libraries, such as CHOMBO [16], Dendro [30], and p4est [17, 18]. Among them, only p4est does not have strict modularity restrictions. Therefore, in this paper, the open-source library p4est [17, 18] is employed in the in-house CABA solver.

**Fig. 5:**
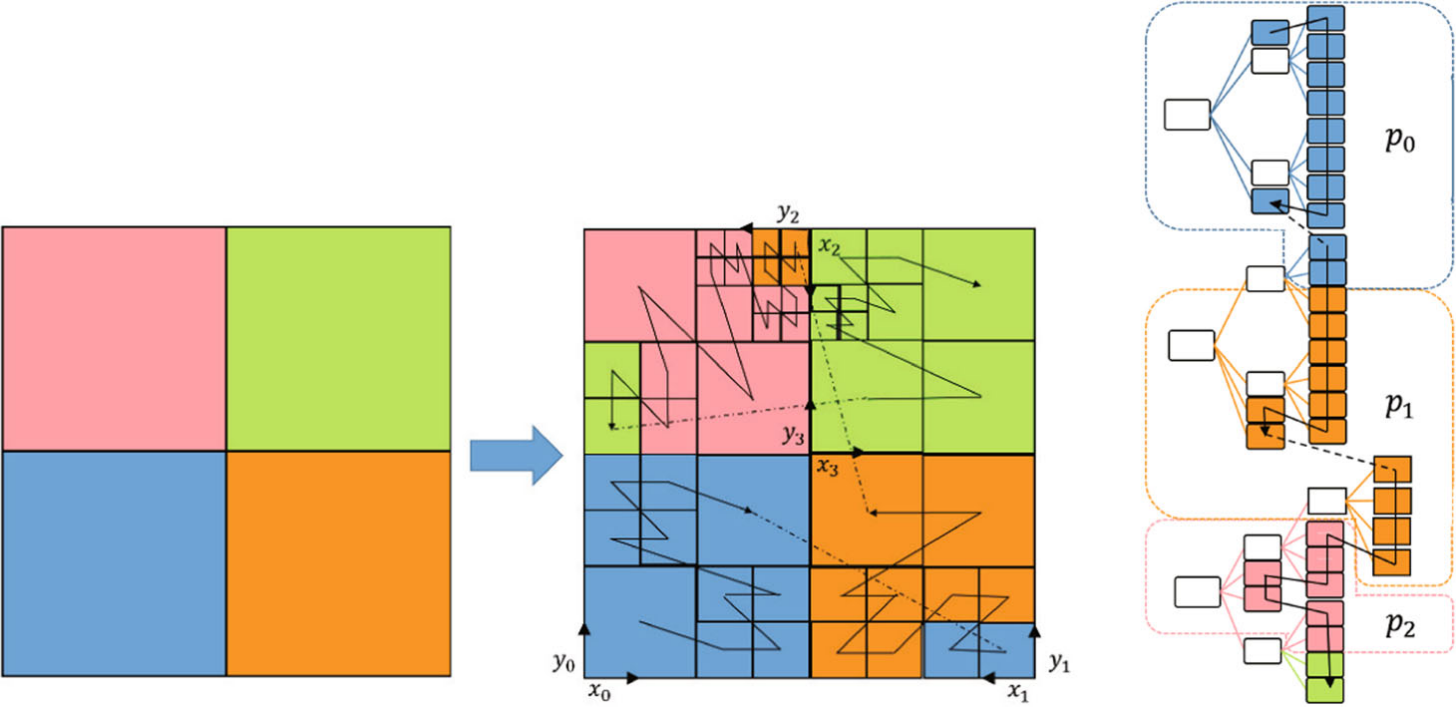
Left: Z-order curve traversal of the quadrants in four trees with different orientation of the forest and partition into four processes which are distinguished by different colors. Right: the corresponding representation of the domain using a quadtree

In the solver, multiple original trees represent a discretization of the physical space *Ω*. The trees define a macro layer, their refined cells define a micro layer, and these two layers make up the domain. The data in the domain is stored in linear tree structure, which is determined by Z-order curve (a space-filing curve). The property of all kinds of space-filing curves, which is called compactness, makes the continuity along the space-filing curve index equal to the continuity in the Cartesian grid. Thus, the Z-order curve could provide an efficient way of partitioning data for load balancing. Meanwhile, it can help to number the nodes by managing the data memory layout in p4est. As shown in Fig. 5, the Z-order curve covers both the macro layer and the micro layer, which means a one-to-one mapping from the spatial coordinates to the index in linear tree storage. And it also shows the order of the index and load balancing between processes (different colors mean different processors).

### Ghost-cell method

**Fig. 6:**
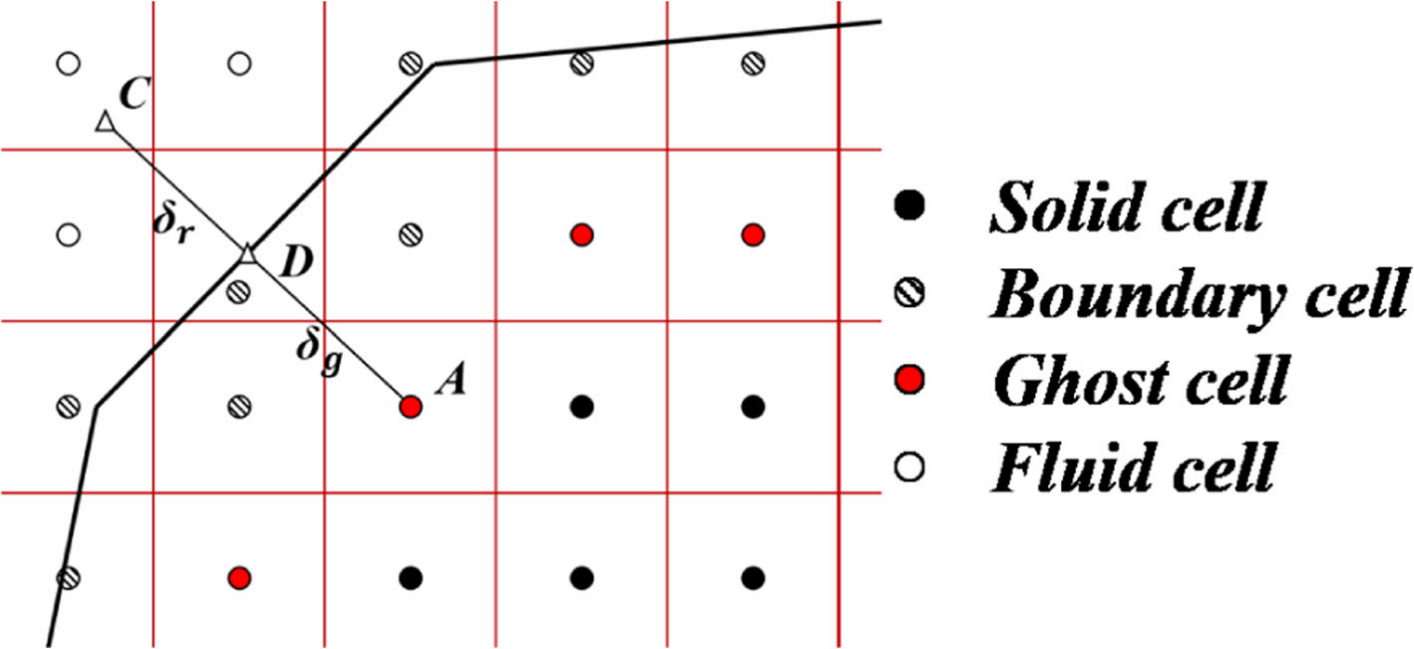
2D schematic of ghost-cell method

For Cartesian grid, the immersed boundary method is generally combined to simulate flow problems because the grid lines are not always aligned with the body [5, 6]. Figure 6 shows a schematic diagram of the ghost-cell method in a two-dimensional case. For closed curves, the Cartesian grids are classified into three categories by the ray-tracing technique [31]: flow cell which is completely inside the fluid, boundary cell which intersects with wall boundary, and solid cell which is completely inside the solid. And the primitive variables of the ghost-cells are determined by variables on the symmetric point.

For example, the symmetric point of ghost-cell *A* is on the extension line of *AD*, where *D* is the closest point on the body surface from *A*. Then *C* is the symmetric point of cell *A* by symmetry, and the primitive variables at the symmetric point are interpolated from the located cell. By using a first-order pressure extrapolation, the wall pressure is taken as the value associated with the nearest cell center, which means the normal pressure gradient is zero. Therefore, the relationship can be expressed as:

7 $$ P_{A}=P_{D}=P_{C}, \rho_{A}=\rho_{D}=\rho_{C},  $$

where *P*_*D*_ and *ρ*_*D*_ represent the wall pressure and the wall density of the point *D*. Then the classical non-penetration and slip wall boundary conditions are considered for inviscid flow, the following equations can be obtained:

8 $$ V_{t,A}=V_{t,C}, V_{n,A}=-V_{n,C},  $$

For viscous flow, non-slip wall boundary condition is considered and the equations are:

9 $$ V_{t,A}=-V_{t,C}, V_{n,A}=-V_{n,C},  $$

Then, a relationship between variables of ghost-cell *A* and symmetric point *C* is established [7, 12]. It needs to be stated that for the high Reynolds number compressible flow, the wall function method is needed to deal with the boundary conditions of the object surface. This part is under development so far and will not be introduced in this article.

**Fig. 7:**
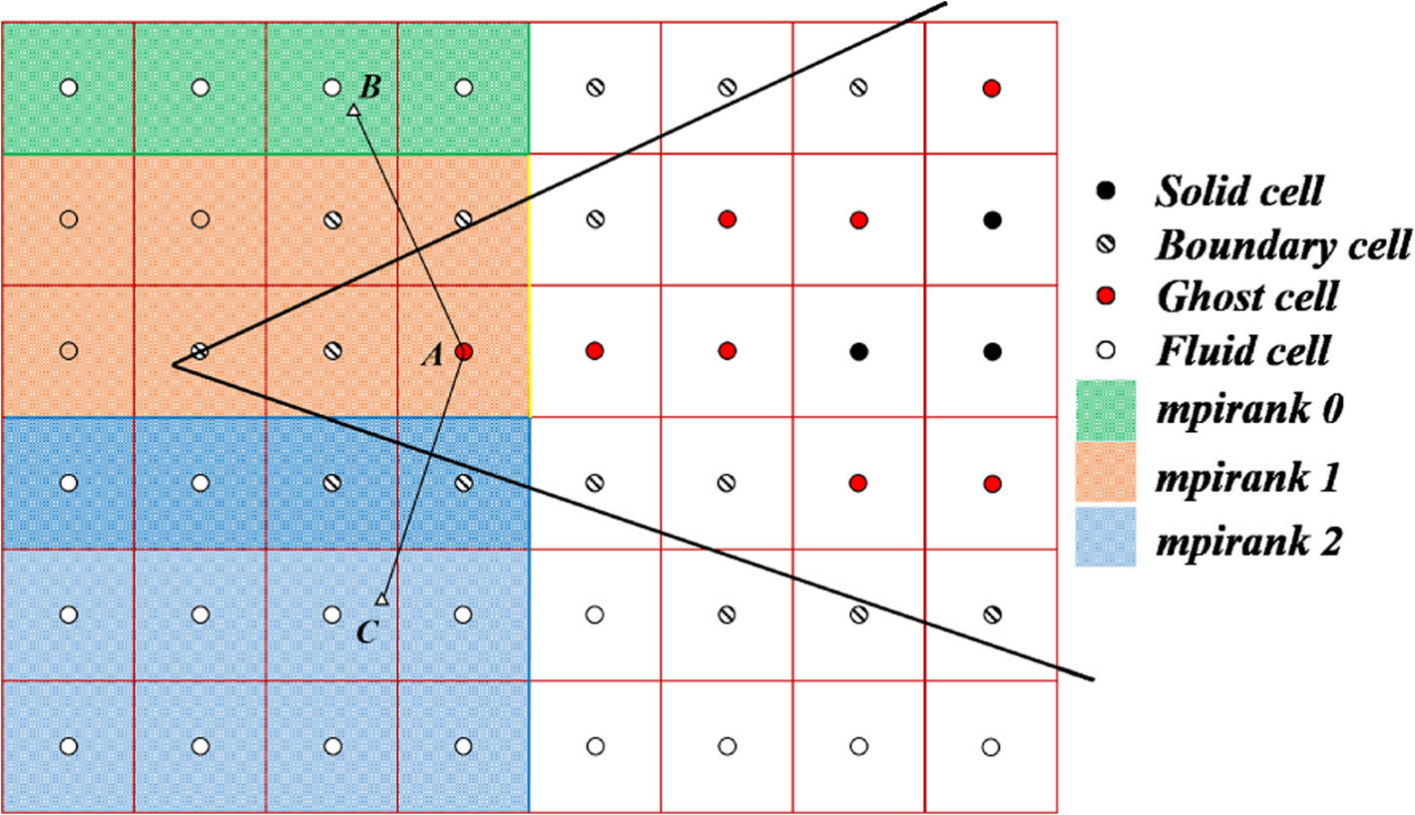
Multi-valued ghost-cell near a sharp leading edge (different color areas indicate a possible partition situation)

In particular, it needs to be emphasized that there will be some challenges when implementing the GCM method under the MPI parallel framework. One is that establishing the relationship between ghost-cell and symmetry point may become very difficult due to distributed storage. As shown in Fig. 7, ghost-cell *A* and symmetric point *B* are not in the same process, and even point *B* is beyond the ghost layer used for general parallel communication. In fact, for a three-dimensional grid of tens of millions, one hundred thousand ghost-cells might not be able to find the symmetric points in the process. Especially for three-dimensional situation, just increasing the ghost layer to two layers can not satisfy all possible symmetric point distributions, let alone the greatly increased communication cost. The second challenge is that the multi-valued ghost-cell method [22] used to handle thin objects requires additional sets of data to be stored in ghost-cell. However, this approach will increase the size of the structure of all cells several times. This means that while greatly increasing the communication costs, only part of the delivered information around the thin object is useful.

For the first challenge, in order to efficiently transfer information between ghost-cell and symmetric point after parallelization, we established a special point-to-cell relationship for each group of ghost-cell and symmetric point that are not in the same process. As long as the grid does not change, these relationships will not change, so the information can be delivered efficiently. The specific process is as follows:

- Collect all the symmetric points that can not be found locally and search them globally. The coordinates of these points need to be temporarily shared globally.
- Establish unique relationships between the original processes and the processes where the symmetric points are located. For a three-dimensional grid of tens of millions, there might be thousands of relationships that need to be established for each process.
- Dynamically apply for storage space for the information to be sent and received according to the established relationship.
- Connect the symmetric points, ghost-cell and these storage spaces through the pointer.

In this way, only one simple communication is required for each time step, and all the information required by GCM is available. But we still have to face the unsteady problem involving complex flow, which requires AMR. The continuously change of the grid means that the above relationships need to be rebuilt frequently, which requires frequent search for points. Thus, in order to reduce calculation costs, we optimized the follow-up point-finding logic. Because in CABA, recursive mesh refinement is prohibited during iteration, each adaptation will not cause drastic changes in the mesh partition. The ID of the process of the information source received during the first point finding is recorded. And searching for these “neighboring processes" in each subsequent point finding process could effectively reduce the calculation cost.

Then, the second challenge encountered when applying GCM to parallelization is discussed. It has always been the difficulty of GCM to treat thin bodies such as the trailing edge of the airfoils and the leading edge of the delta wing. If the thickness of a body becomes smaller than 1.5 times of cell size, some ghost-cells have to handle both sides of the body as shown in Fig. 7. Cell A inside the geometry is the ghost-cell for the upper side of the corner surface with symmetric point B, as well as for the lower side with symmetric point C. Ignoring the multi-value points will cause unavoidable errors in the flow simulation of shapes such as delta wing. A multi-valued ghost-cell [22] is usually employed to handle this problem. By sweeping in the three coordinate directions, the ghost-cell A could have sets of properties computed from both sides of the trailing edge. In a three-dimensional problem, a ghost-cell may have 3, 4 or even more symmetric points. This method needs to open up storage space for all possible data of all ghost-cells, which will greatly increase the cost of parallel communication. And only a few of the additional information will be used in the simulation of the flow field near the multi-value point.

In order to enhance the accuracy and robustness of the algorithm in calculating three-dimensional thin shape, the multi-valued method is improved. We collect the intersecting surfaces of all surrounding boundary cells and search the symmetric point for each surface. By matching the vectors from the center of the cell to the symmetric point with the normal vectors of the cell surfaces, each ghost-cell can match up to 6 symmetric points in the three-dimensional case. The information of the local symmetry point can be accessed by pointer, and the information of the symmetry point of other processes will be passed through the point-to-cell relationship mentioned above.

In this way, CABA can get as much information as possible to fit the surface of the object when simulating the flow field. Compared with non-special processing of multi-value points, this method can guarantee the authenticity of the flow simulation near the thin object. This is of great significance for dealing with three-dimensional pointed objects such as supersonic wave riders.

## Numerical results and discussions

In this section, several representative numerical cases are tested to verify the performance of the developed in-house solver on adaptive Cartesian grid. Specifically, it includes the problem of two/three dimensional viscous oblique shock-mixing layer interaction, inviscid/viscous flow around a single NACA0012 airfoil and two staggered NACA0012 airfoils, unsteady viscous flow past one cylinder, three-dimensional inviscid transonic flow over ONERA M6 wing, three-dimensional viscous flow around a sphere, laminar flow around delta wing, etc.

### Shock-shear layer interaction

#### Two-dimensional case

This test is carried out to evaluate the ability of the AMR technique to capture the small-scale vertical structures interacting with a shock discontinuity [32, 33]. A series of vortices are generated by a spatially developing mixing layer, and interactions appear between the downstream vortices and the shock which is reflected by a (slip) wall at the lower boundary. The initial conditions are shown in [33], and the mixing layer is developed by the following hyperbolic tangent profile:

10 $$ u=2.5+0.5\tanh{(2y)},  $$

11 $$ v'=\sum_{k=1}^{2} \alpha_{k}\cos{(2\pi kt/T+\phi_{k})}exp(-y^{2}/b),  $$

where period *T*=*λ*/*u*_*c*_, wave length *λ*=30,*b*=10,*a*_1_=*a*_2_=0.05,*ϕ*_1_=0,*ϕ*_2_=*π*/2, and the convective velocity *u*_*c*_=2.68. The rest of the boundary conditions can refer to [32], which will not be repeated here. The Reynolds number is 500 for this case.

**Fig. 8:**
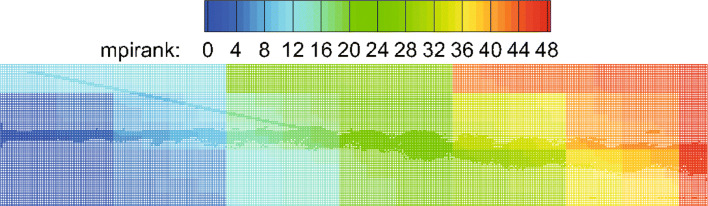
Adapted mesh at the final time *T*=120 (colored by the process number)

**Fig. 9:**
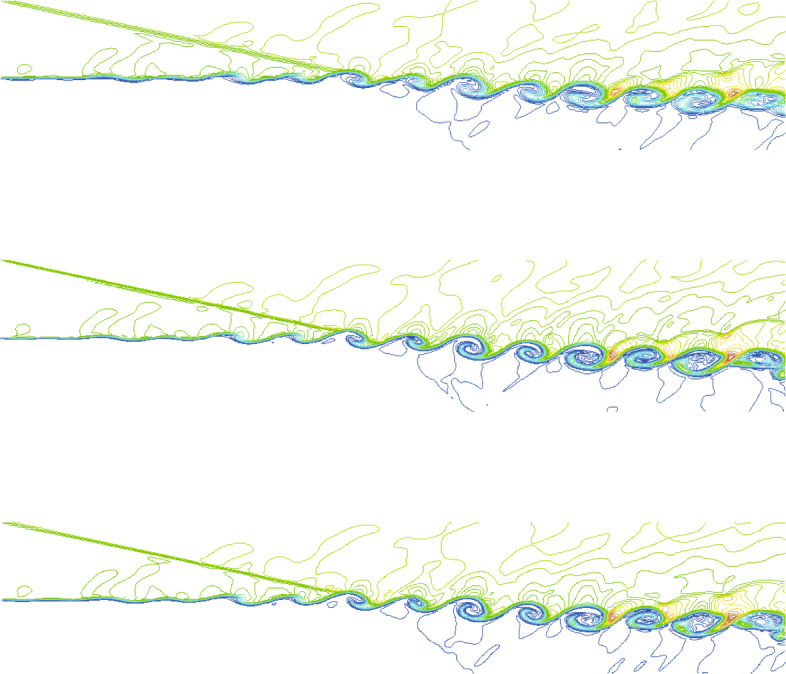
Density contours of two-dimensional viscous shock-mixing layer interaction problem. *T*=120. Top: coarse uniform grids (400×80). Middle: fine uniform grids (800×160). Bottom: adapted grids based on coarse grid

The computational domain is [0,200]×[−20,20], which is divided into 400×80 initial cells. For this unsteady problem, a solution-based refinement is carried out every time step. The divergence and curl of velocity criterion are both used in this example because it contains both shock and vortex structures. In Fig. 8, the adapted mesh at the final calculation time *T*=120 is shown, which is colored by the process number (i.e., different colors represent different processes). It is illustrated from the figure that the key flow characteristics in the flow field (whether it is a shock wave or vortex structures) can be effectively captured by the criterion in our CABA solver. The result also shows the excellent dynamic partitioning ability and load balancing performance of CABA for dynamic adaptive grid. Moreover, the calculated density contours at the final calculation time are presented in Fig. 9 and compared with the numerical results obtained by the coarse uniform mesh (i.e. 400×80 cells) and the fine uniform mesh (i.e. 800×160 cells). It can be seen that the vortex structures downstream of the shock wave obtained by the coarse uniform mesh have obvious dissipation compared to the results of adaptive grid and the fine uniform grid. Therefore, the parallel CABA solver developed in this paper can obtain similar results with the fine meshes by using fewer grid cells.

#### Three-dimensional case

**Fig. 10:**
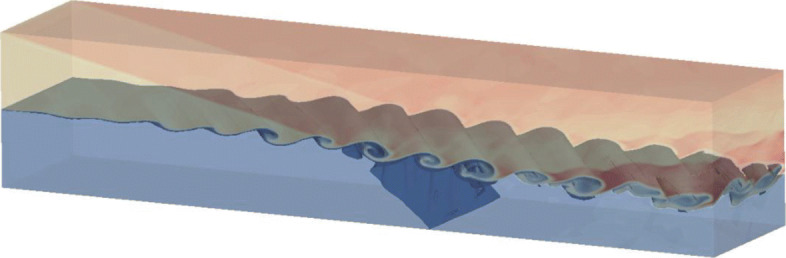
Density contours of three-dimensional viscous shock-mixing layer problem on adaptive Cartesian grids. *T*=120

Next, we consider the three-dimensional shock-mixing layer interaction problem as in Ref. [33]. The computational domain is to stretch a two-dimensional surface along the *z*-direction, with symmetrical boundary conditions applied at both ends. Considering the three-dimensional effect, the inflow conditions (12) are modified as:

12 $$ v'=\sum_{k=1}^{2} \alpha_{k}\cos{(2\pi k(t/T+z/L_{z})+\phi_{k})}exp(-y^{2}/b),  $$

where *L*_*z*_=40 is the length in the *z*-direction. The density contours with iso-surfaces at *t*=120 are shown in Fig. 10. Due to the three-dimensional disturbance, a phase difference occurs and the spanwise vortex structure develops regularly along the *z*-direction. After the first shock hits the layer, the vortex structure in the *z*-direction deforms significantly. Then, the shock is reflected by the boundary and strikes the layer again, which makes the interaction of vortex further developed.

**Fig. 11:**
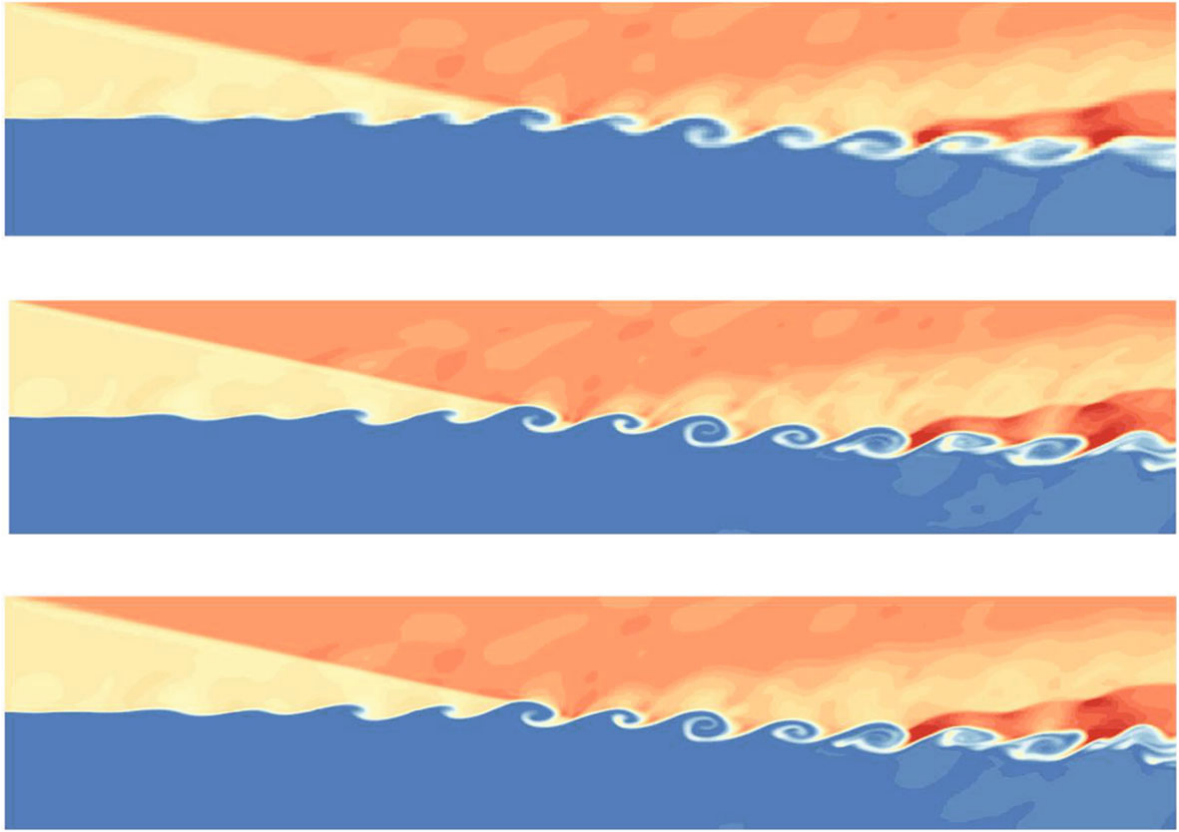
Density contours on three kinds of grid at *z*=20 plane. *T*=120. Top: uniform coarse grids (400×80×80). Middle: uniform fine grids (800×160×160). Bottom: AMR with one refinement level based on the coarse grid

To better represent the performance of the developed parallel adaptive Cartesian solver, the density contours at location *z*=20 plane are shown in Fig. 11. Analogy to the analysis of two-dimensional case, the comparison with the results simulated from uniform grid is also given in Fig. 11. It can be found that the adaptive meshes have similar accuracy compared to the fine meshes, while the results on coarse meshes are the most dissipative. It is worth mentioning that the number of grids with AMR technique is about 25.6% of that using the fine grid at the final simulation time *t*=120, while the calculation cost is only 1/6 of that.

### Flow around NACA0012 airfoil and two staggered NACA0012 airfoils

#### Inviscid transonic flow around NACA0012 airfoil

**Fig. 12:**
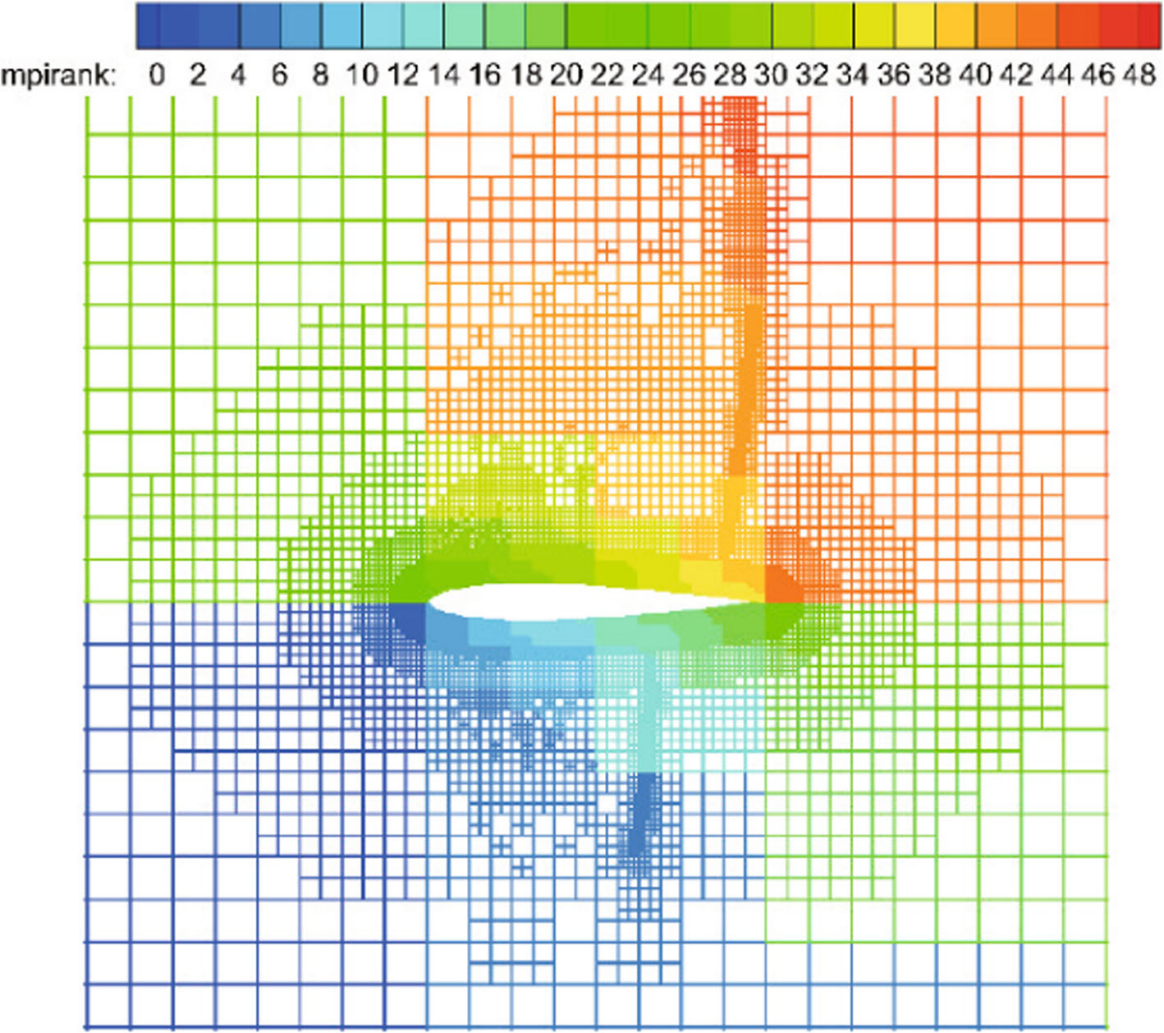
Adapted mesh for the problem of inviscid flow over a single NACA0012 airfoil (colored by the process number)

**Fig. 13:**
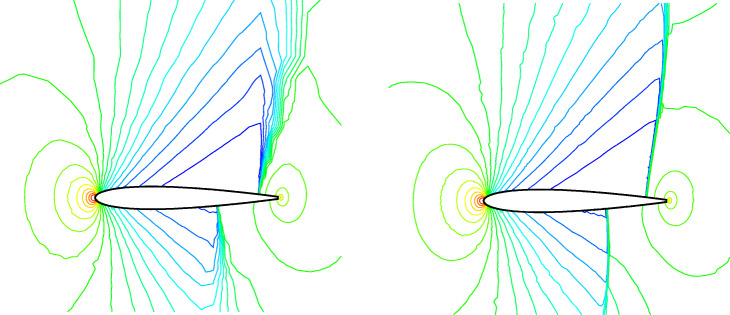
Pressure contours for the problem of inviscid flow over a single NACA0012 airfoil. Left: without solution-based refinement. Right: with solution-based refinement

**Fig. 14:**
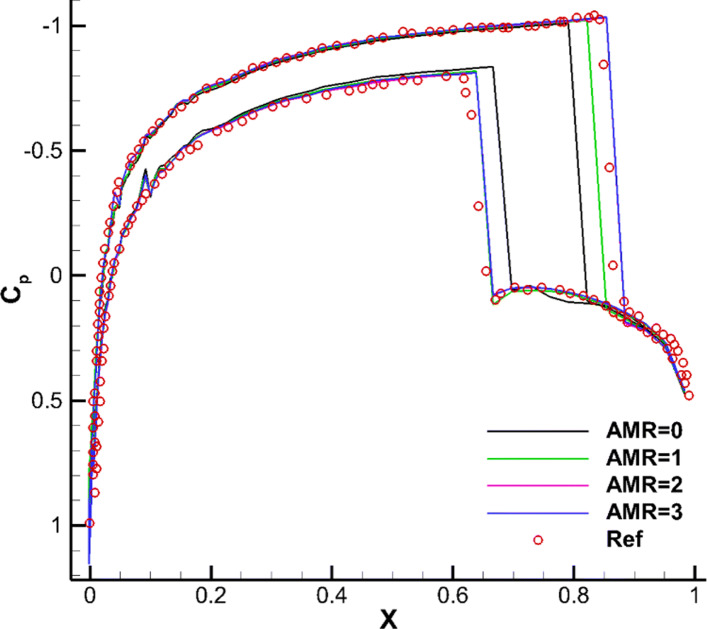
Simulated surface pressure coefficient distributions obtained with different levels of refinement for the inviscid flow over a single NACA0012 airfoil

The transonic flow around a single NACA0012 airfoil is selected here to verify the ghost-cell method of the CABA solver in this paper. First, a classical inviscid case with Mach number *Ma*=0.85 and angle of attack *α*=1^∘^ is tested. The computation domain is [0,32*c*]×[0,32*c*], where *c* is the chord length of airfoil. The initial mesh (64×64) is first refined six times near the body boundary, and the number of grids is 15,070. Then three levels of solution-based refinement are carried out by 48 cores in parallel during the computation, with the final number of the adapted grid 23,119. Figure 12 shows the grid partition in the steady-state, and it can be seen that the strong shocks are well captured on both upper and lower surfaces. Figure 13 shows the pressure contours under three times solution-based refinement and the results without solution-based refinement for comparison. It can be clearly seen that the shock is smeared without solution-based refinement, while the result after adaptive processes is sharper. Additionally, the grid independence analysis is carried out, with the main aim to verify the obtained simulations under the mesh conditions with different levels of *h*-refinement. The pressure coefficient *C*_*p*_ is shown in Fig. 14. The figure gives a comparison of the results with different AMR times and the results of the body-fitted grid with a mesh number of 20,480 in AGARD [34]. It can be found that the present results compare reasonably with the results of AGARD and AMR can capture the shock wave position more accurately and meanwhile reduce the oscillation of *C*_*p*_ to a certain extent.

#### Inviscid supersonic flow around two staggered NACA0012 airfoils

Compared with the single block-structured grid, one of the main advantages of the Cartesian grid is that it can directly generate corresponding grids on multiple objects for numerical simulation. Therefore, a supersonic flow around two staggered NACA0012 configuration with Mach number 2 and the attack of angle 0 is considered. The two airfoils are staggered by half a chord length in the pitchwise as well as chordwise direction. The computation domain is [0,32*c*]×[0,32*c*], where *c* is the chord length of airfoil. The initial mesh (64×64) is first refined seven times near the body boundary. Then three levels of solution-based refinement are carried out by 48 cores in parallel during the computation, and the final number of the adapted grid is 33,362.

**Fig. 15:**
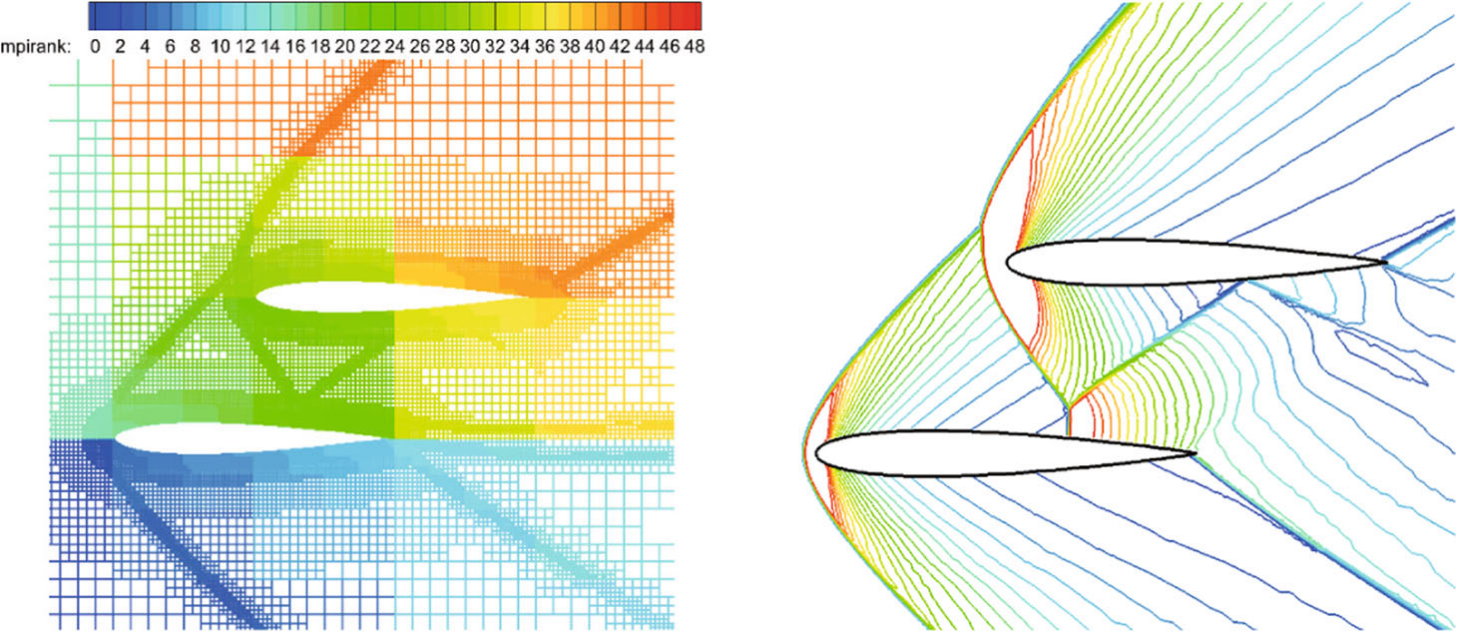
Supersonic flow around two staggered NACA0012 airfoils. Left: the partition of adaptive grids on 48 cores. Right: pressure contour

**Fig. 16:**
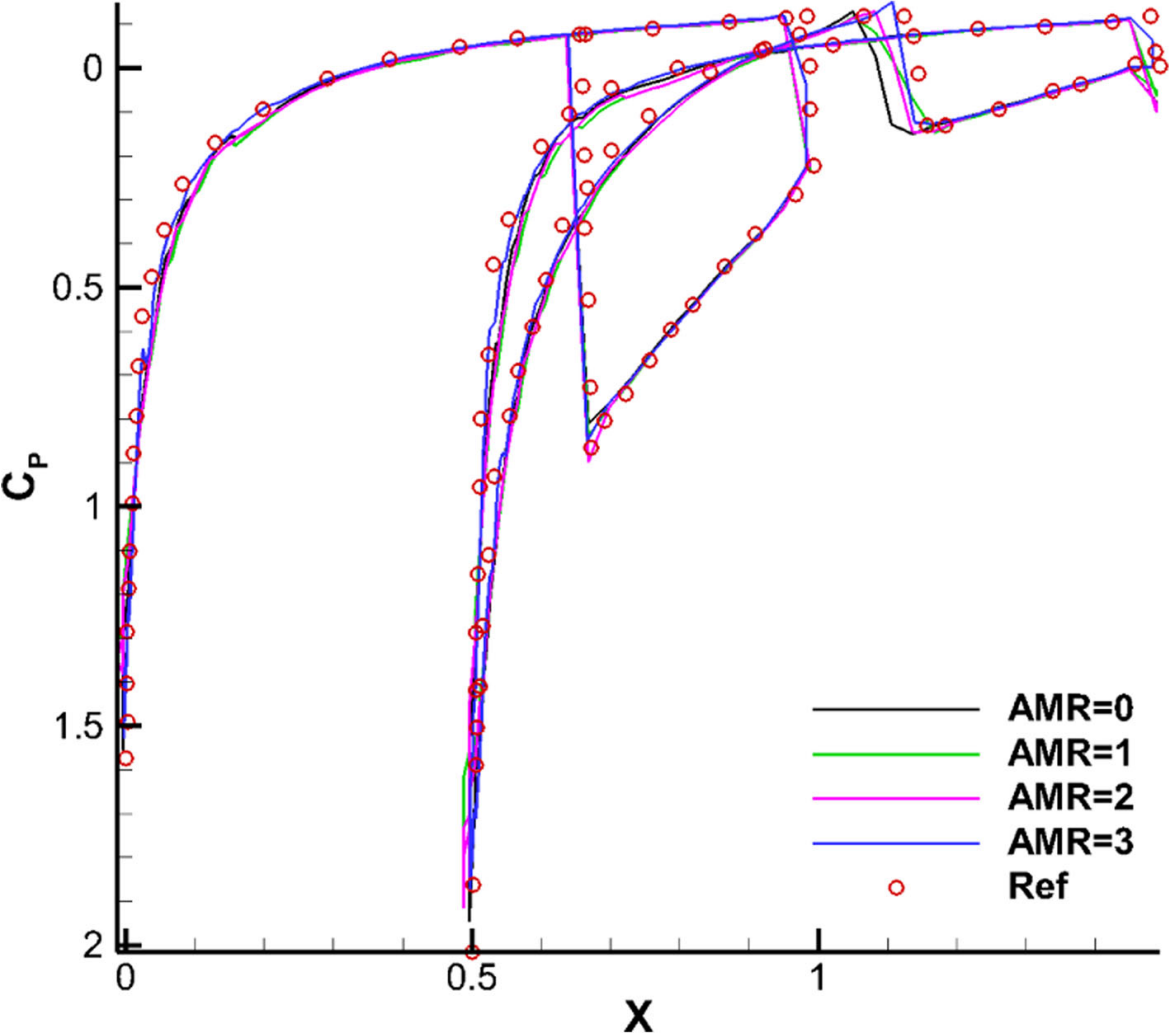
Simulated surface pressure coefficient distributions obtained with different levels of refinement for the inviscid flow over two staggered NACA0012 airfoils

For this test case, we mainly focus on the complex shock structures (such as the bow shock, reflected shock, trailing edge shock, etc.) and their interactions. Figure 15 shows the partitioning of the adaptive Cartesian grid and pressure contours in the steady state. It can be clearly seen that the CABA solver accurately captures the leading edge shock, the trailing edge shock, and the reflection of shock between the two airfoils of this problem. The pressure coefficient *C*_*p*_ is shown in Fig. 16. It can be seen that the results with three levels of solution-based refinement match well with the results of the high-order DG method on adaptive grid [14]. It is worth mentioning that AMR has played an obvious role in capturing the reflected shock wave on the lower surface of the top airfoil.

#### Viscous transonic flow around two staggered NACA0012 airfoils

**Fig. 17:**
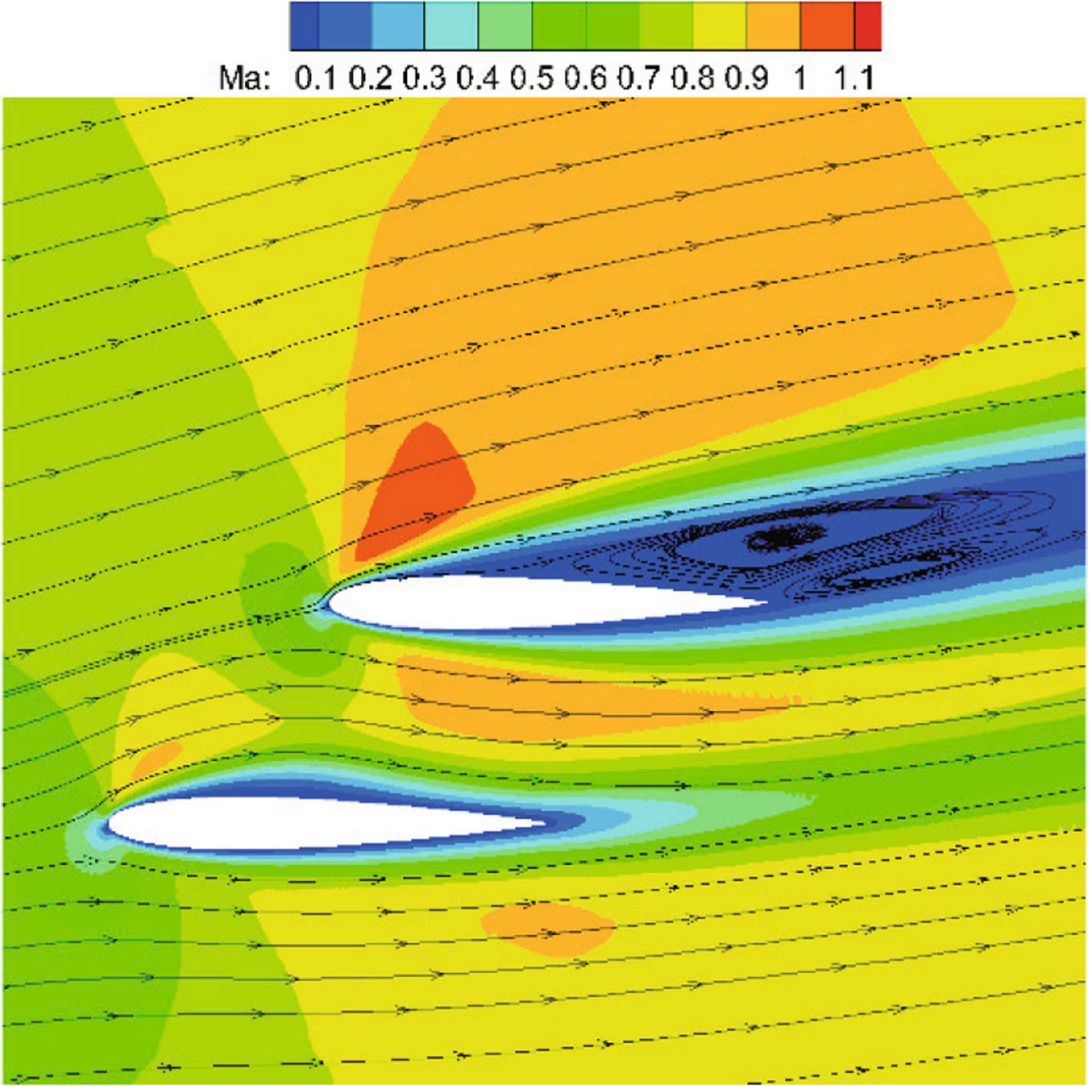
Mach number contours with streamlines and separation around the top airfoil

**Fig. 18:**
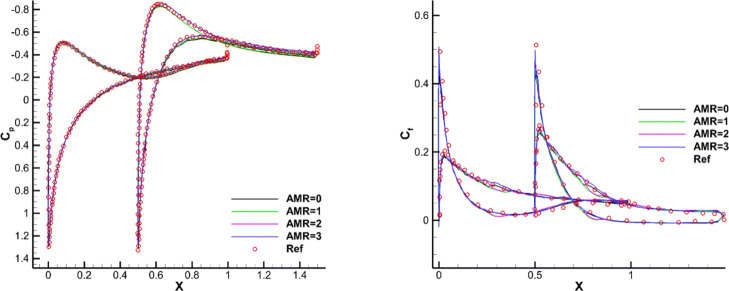
Simulated results obtained with different levels of refinement for the viscous flow over two staggered NACA0012 airfoils. Left: pressure coefficient. Right: skin-friction coefficient

To further demonstrate the accuracy of CABA, flow over two staggered NACA0012 configurations with *Ma*=0.8,*Re*=500, and angle of attack *α*=10^∘^ is simulated. In this case, prominent vortexes extend over 50% of the chord on the upper surface of the upper airfoil and a large separated region appears. The length of initial grid in this case is set to 0.25*c*, while the computation domain remains the same as the previous inviscid case. The initial mesh is firstly refined six times near the airfoil surface, then three times of solution-based refinement are carried out during the time evolution to correctly capture flow evolution. Figure 17 presents the simulated streamlines and it can be clearly seen that two vortices are generated in the separated region on the upper surface of the top airfoil. Figure 18 shows the corresponding distributions of pressure coefficient *C*_*p*_ and skin-friction coefficient *C*_*f*_. The simulated pressure coefficient and skin-friction coefficient are also compared with the results from unstructured grids [35]. It shows good agreement between presented results and reference data. It also can be clearly seen that the oscillation of *C*_*f*_ is greatly reduced after solution-based refinement.

### Von Karman vortex street

**Fig. 19:**
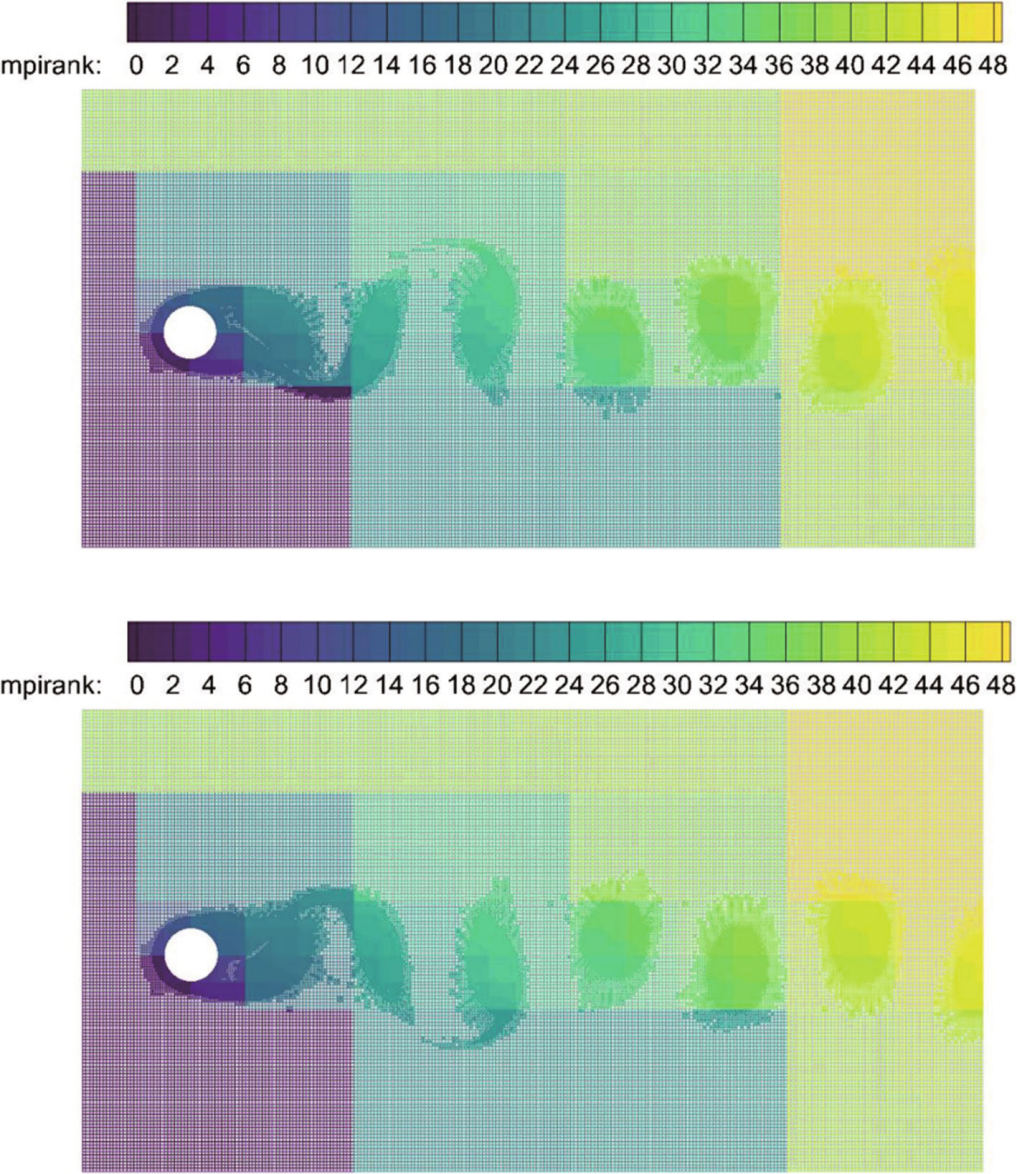
The dynamic partition of adaptive grids at two different times on 48 cores

**Fig. 20:**
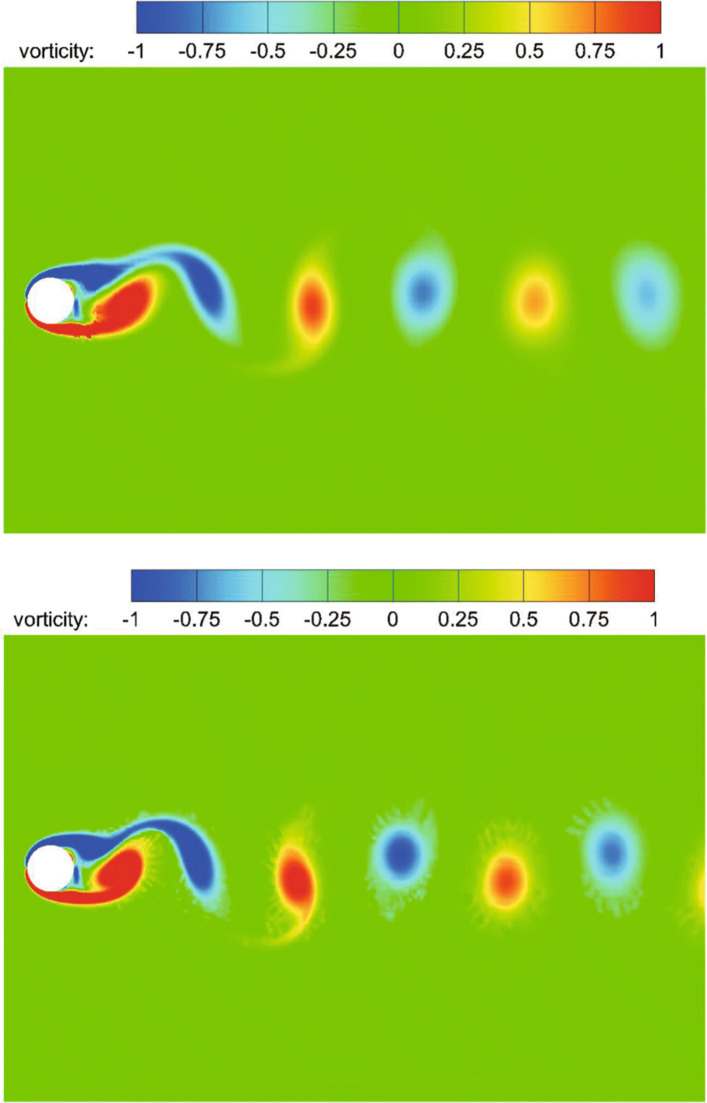
Instantaneous vorticity contours for flow around a cylinder. Top: without solution-based refinement. Bottom: with three times solution-based refinement

The classical problem of flow past a circular cylinder is considered, which has been extensively studied both experimentally and numerically [36]. Considering its highly unsteady characteristics, this example is also used to verify the performance of the CABA solver’s automatic partitioning algorithm for adaptive Cartesian grids. In the present calculation, the Reynolds number is set to 200 based on the inflow velocity and the cylinder diameter, and the free-stream Mach number is 0.3. The computation domain is [0,20*D*]×[0,10*D*], where *D* is the cylinder diameter. The initial mesh (320×160) is first refined six times near the body boundary to better describe the profile of the cylinder. Then three levels of solution-based refinement are carried out by 48 cores in parallel during the computation, and the final number of the adapted grid is around 261,338. Figure 19 shows the dynamic partition of the adaptive Cartesian grid at different times. It can be clearly seen that the CABA solver in this paper has reasonable dynamic partitioning capabilities for the adaptive Cartesian grid. The vorticity contours without solution-based refinement and with three times solution-based refinement are both given in Fig. 20. The evolutions such as the generation, shedding, and periodic change of vortex street are well captured in both cases. It can be seen that a longer distance of vortex evolution can be realized with the combination of adaptive technology, while the vortex structures have significantly greater dissipation at downstream without AMR. Table 1 compares the lift coefficient *C*_*L*_, drag coefficient *C*_*D*_ and Strouhal number with other numerical results obtained by unstructured grids with AMR [36] and Cartesian grid [37]. It can be seen that *C*_*L*_ and *C*_*D*_ compare well with references and the predicted Strouhal number is 2.3-4.7% larger than the literature results.

**Table 1:** Comparison of the present results against reference for flow over a cylinder

	*C*_*L*_	*C*_*D*_	Strouhal number
Ref1 [36]	0.67	1.39	0.192
Ref2 [37]	0.74	1.32	0.196
CABA	0.69	1.43	0.201

### Flow around ONERA M6 wing

**Fig. 21:**
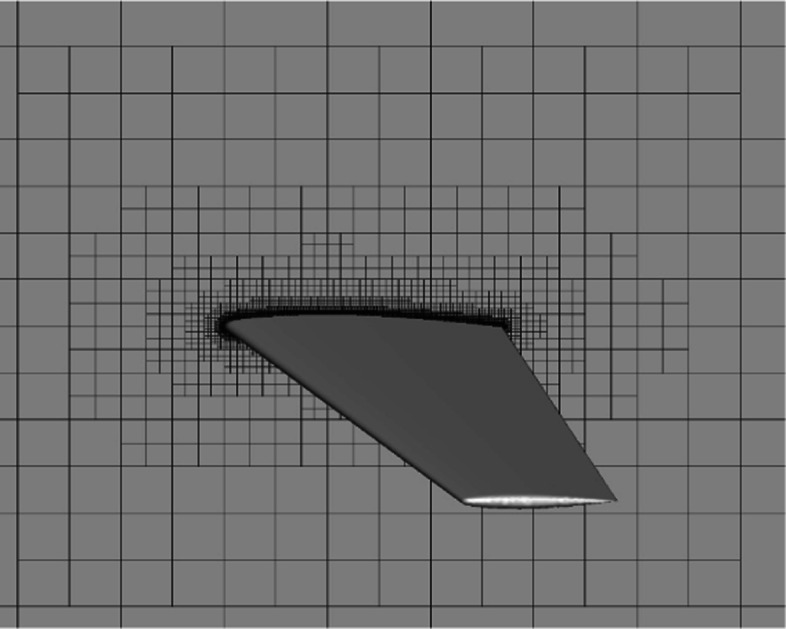
The initial computational grids with geometry-based AMR

**Fig. 22:**
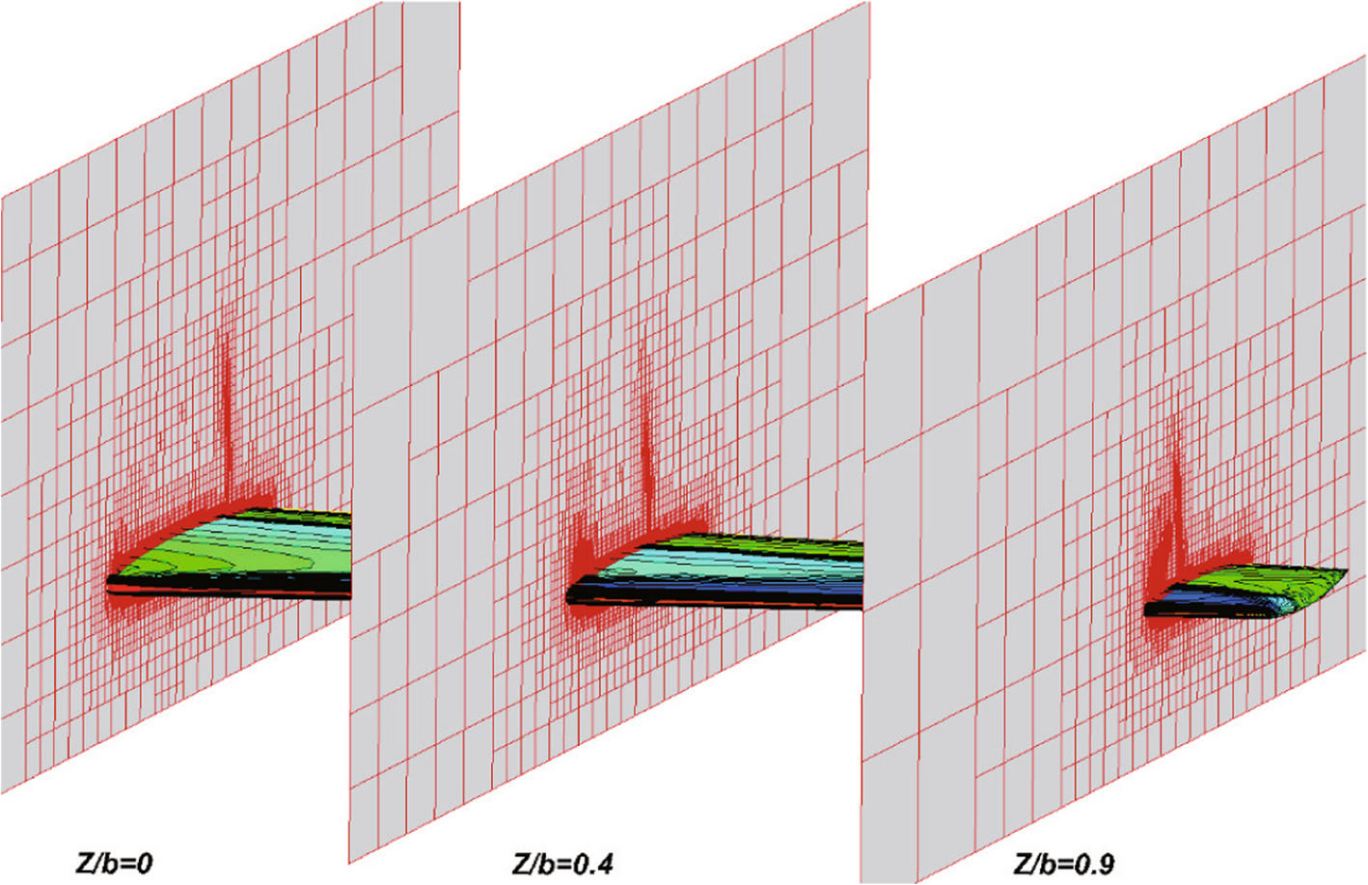
Partial view of the adaptive grids with solution-based AMR (*Z*/*b*=0,*Z*/*b*=0.4,*Z*/*b*=0.9)

In this section, the transonic flow around an ONERA M6 wing is tested to validate the overall performance of the developed CABA solver. For instance, the automatic generation of three-dimensional Cartesian grids, the robustness of the ghost-cell method for three-dimensional cases, and the ability to capture flow field characteristics. The free-stream Mach number is 0.8395 and the angle of attack *α* is 3.06^∘^, with other conditions the same as Ref. [38]. The computational domain is defined as [0,16]×[0,16]×[0,16], and the initial grid step size is set to 1. First, the STL file (consisting of 16280 triangular cells) of the M6 wing is input for the CABA solver. Then, the required Cartesian grid is automatically generated for subsequent calculations. Based on the adaptive Cartesian grid in Fig. 21, three levels of solution-based refinement are carried out during the computation, and the final number of the adapted grid is 7,166,614. Figure 22 depicts the adapted mesh at different span locations, where the grids near the leading edge of the wing and the shock wave region are significantly refined.

**Fig. 23:**
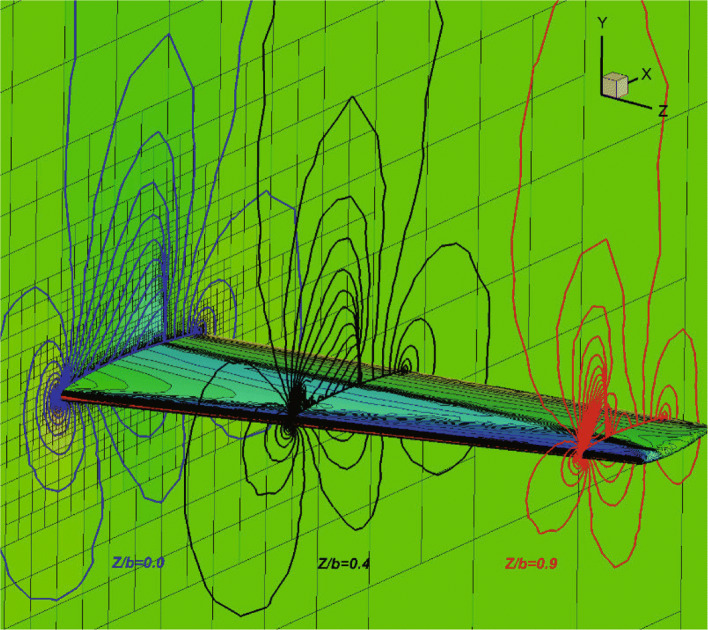
Pressure contours for flow around ONERA M6 wing (*Z*/*b*=0,*Z*/*b*=0.4,*Z*/*b*=0.9)

**Fig. 24:**
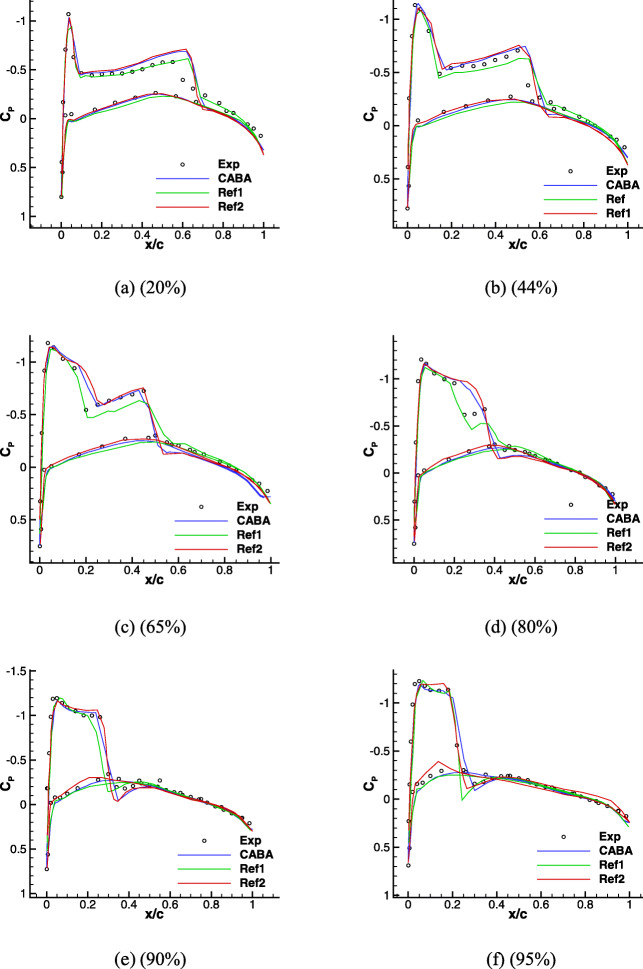
Pressure coefficient distributions on the different spanwise sections of ONERA M6 wing

The pressure contours are shown in Fig. 23 and the “ *λ*" shape shock wave on the upper surface of the wing is clearly captured. Figure 24 shows the pressure coefficient distributions at selected spanwise locations computed by CABA. The experimental results [39] and Euler results getting from high-order methods on unstructured grids reported in [38, 40] are also presented in Fig. 24 for comparison. The results of CABA have well agreement with the computational data, while small deviations compared with experimental data due to the lack of viscous effects.

**Fig. 25:**
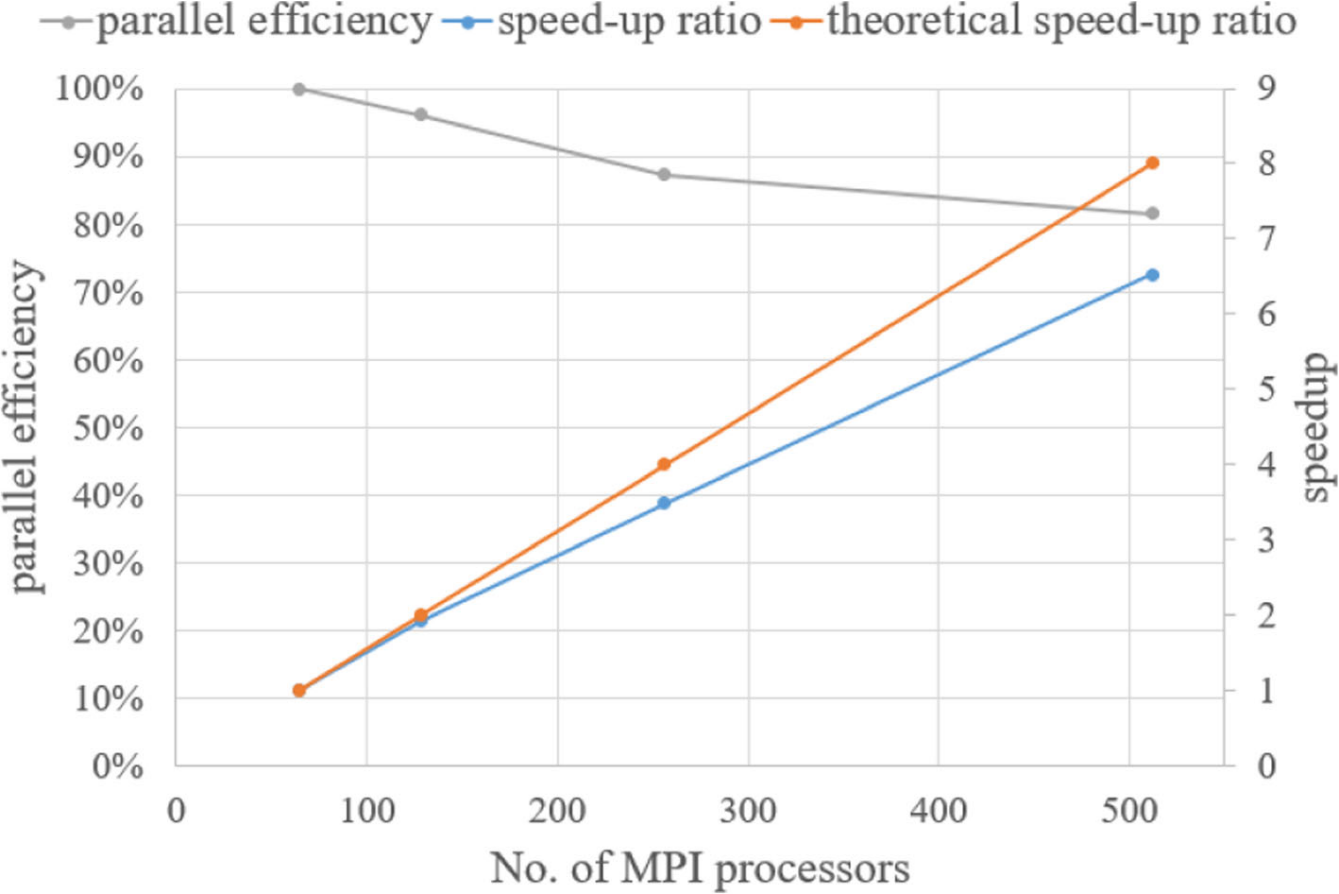
Parallel efficiency and speed-up ratio for different number of processes

At the same time, we also used this case to test the parallel performance of the in-house CABA solver. To ensure enough calculations, the minimum size of the grid in this case is reduced by 1/4, and the final number of grids increases to 44 million. The case was performed on a cluster, where the nodes have two Intel Gold 6149 CPUs at 3.1 GHz per node. Figure 25 shows the parallel efficiency under different number of processes. It can be seen that when the number of processes increases to 512, the parallel efficiency can still be maintained above 80%, which indicates the feasible parallel scale expansion prospect of our numerical solver.

### Flow around a sphere

#### Viscous subsonic case

**Fig. 26:**
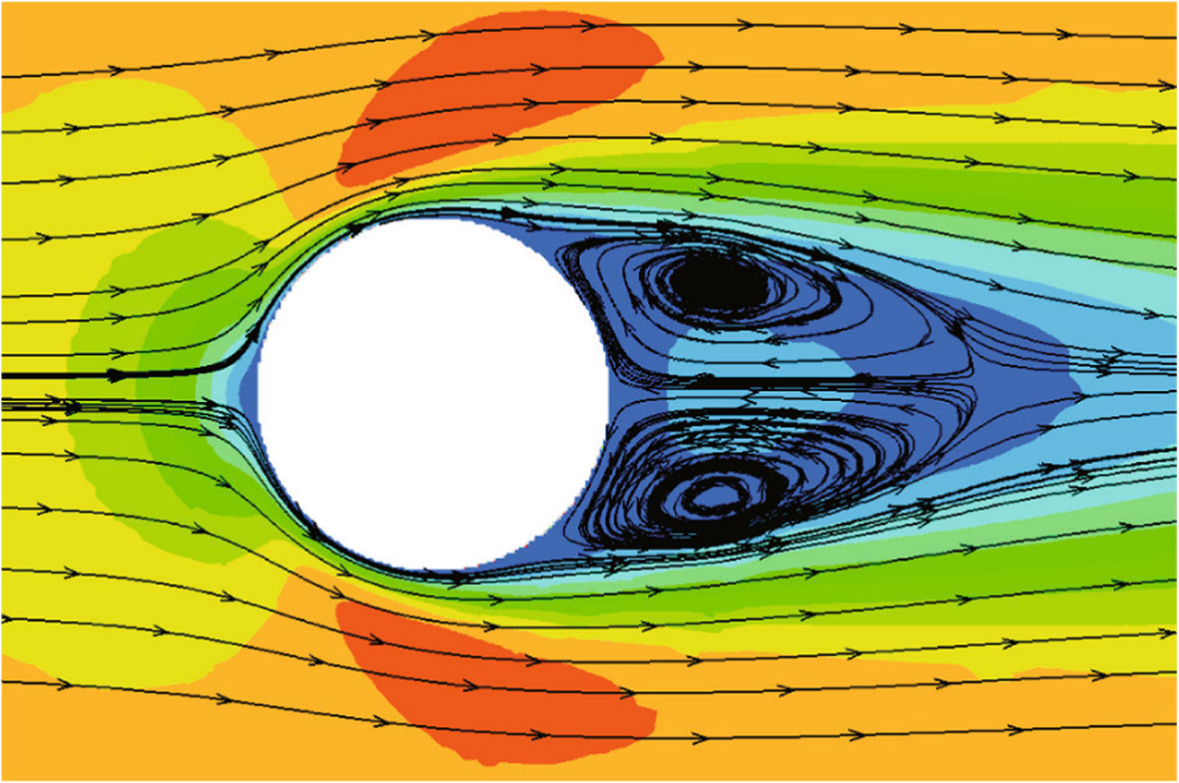
Streamlines and Mach number contours for subsonic flow around a sphere (*Z*=0)

To test the performance of CABA in simulating the 3D compressible viscous flow, the case of the steady viscous flow around a sphere is solved [41, 42]. It was tested on a cluster using one node (128 cores, two Intel Gold 6149 CPUs per node). The Reynolds number based on the diameter of the sphere is 118. The free-stream Mach number is set as 0.2535 following the same experimental condition [43]. The diameter of the sphere is *D*=1, and the computational domain is [0,24]×[0,24]×[0,24]. The initial mesh (24×24×24) is refined seven times near the body boundary to better describe the profile of the sphere and three levels of solution-based refinement are carried out by 128 cores in parallel during the computation. The number of the grid is 1,539,782 and increased to 1,801,220 after solution-based AMR. Figure 26 presents the computed Mach number contours and streamlines. Since the Reynolds number of the flow is less than the critical Reynolds number, the flow is considered steady and a separation bubble appears. As noted in Fig. 26, the wake pattern remains highly symmetric. To quantitatively validate the accuracy of the computed results, the predicted separation angle *θ*, and the length of wake *L* are compared with the results of body-fitted grids [42]. The details are shown in Table 2, where the results agree well with the reference data and experimental data [42].

**Table 2:** Comparison of the present results against reference for subsonic flow around a sphere

	*θ*	*L*
Experiment	151.0	1.07
Ref [42]	125.1	1.32
CABA	130.8	0.97

#### Viscous supersonic case

**Fig. 27:**
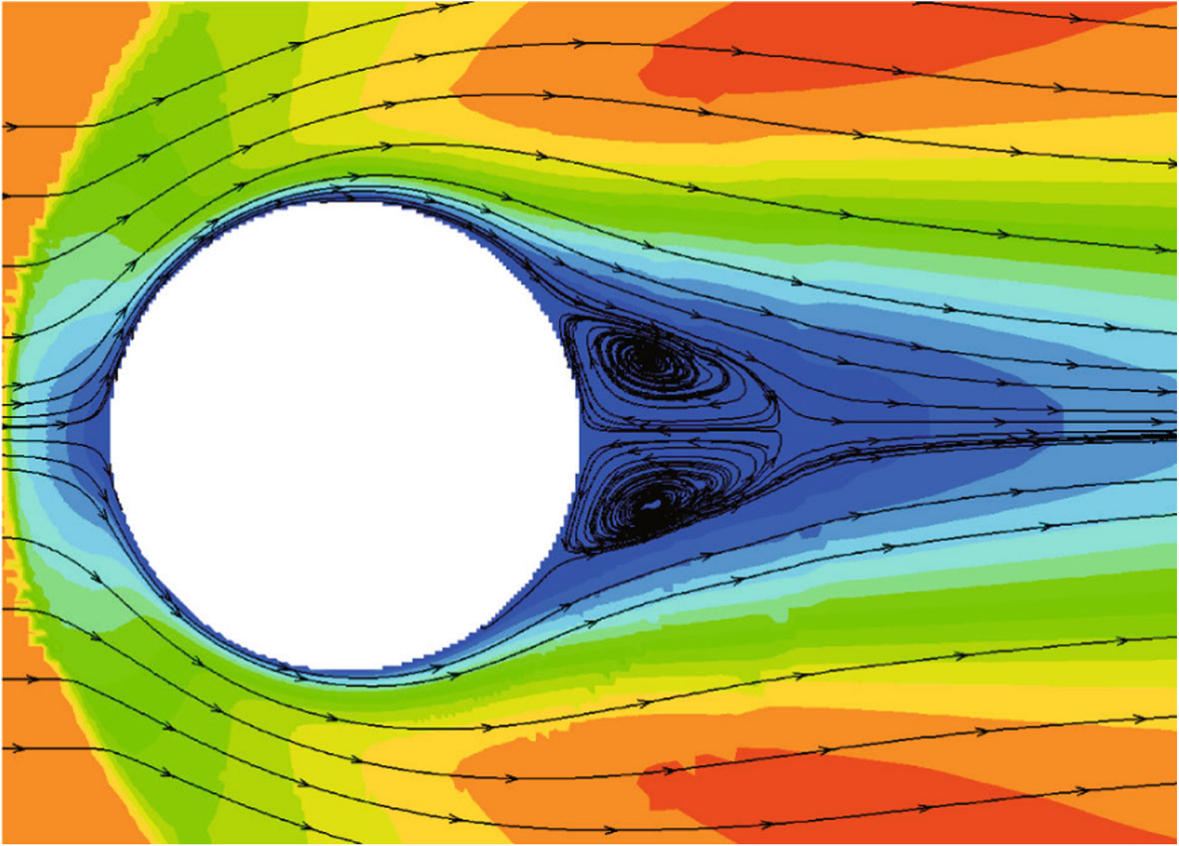
Streamlines and Mach number contours for supersonic flow around a sphere (*Z*=0)

**Fig. 28:**
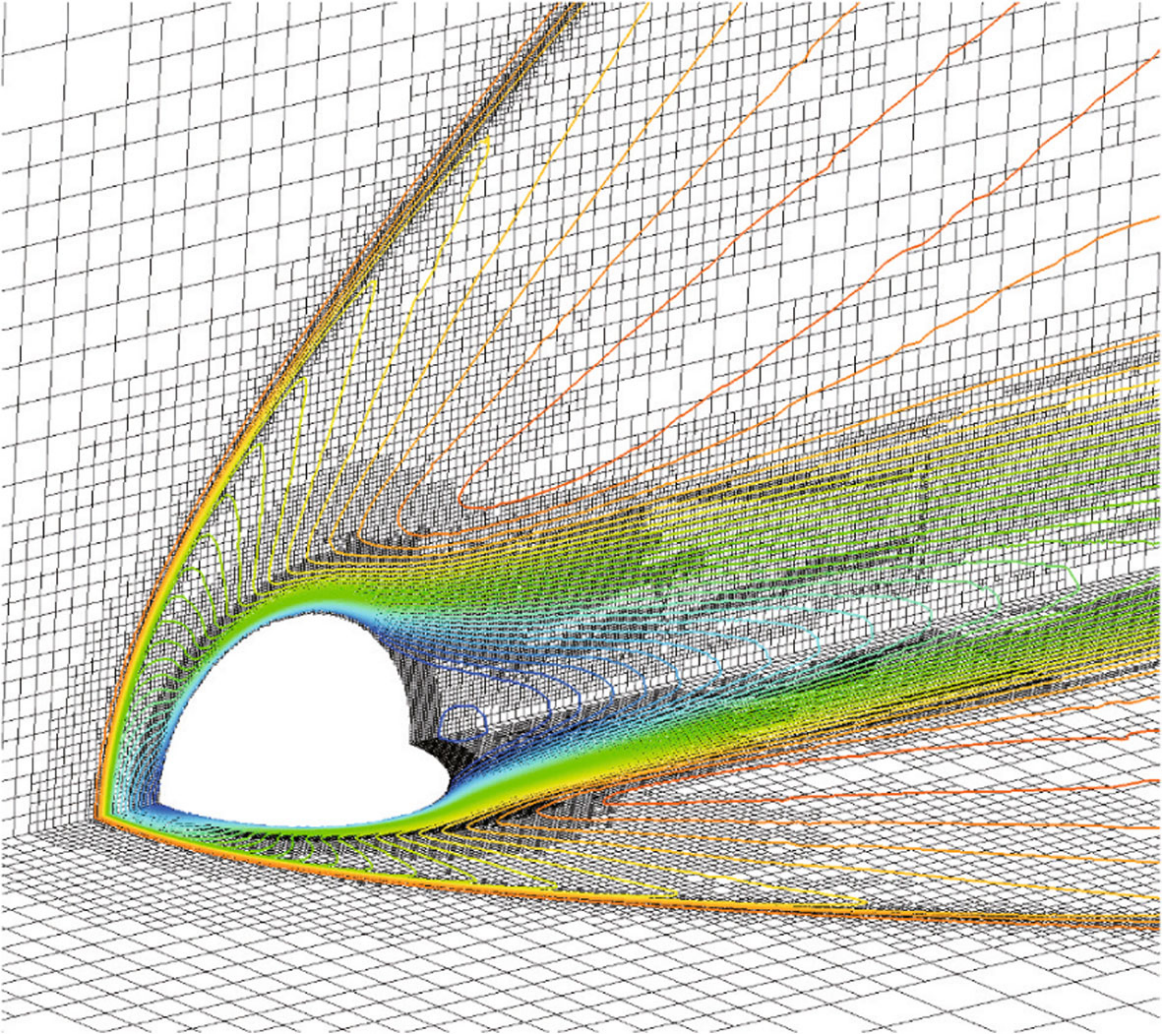
The adaptive grids and Mach contours on two sections

The supersonic flow around a sphere is further tested to assess the capacity of the current method for capturing the shock wave for 3D flows. The Reynolds number based on the diameter of the sphere and the free-stream Mach number are set as 300 and 2 respectively to achieve steady and axisymmetric flow field. The computational domain and initial mesh are the same as the case in Sec. 3.5.1 and the final number of grid is 2,044,980. The hardware conditions are the same as the previous example. Figure 27 depicts the streamlines and Mach number contours, from which stable and axisymmetric flow structures can be clearly observed. The adaptive grid on two sections along with the Mach number contours is shown in Fig. 28. Clearly, the mesh is adequately refined in the shock reflection region and the shock is captured sharply. To quantitatively assess the results, Table 3 compares the shock stand-off distance *L*_*s*_, separation angle *θ*, and wake length *L* computed by body-fitted grids [38, 44]. It can be found that the results are in good agreement. The observations in both Sec. 3.5.1 and Sec. 3.5.2 validate the correctness and robustness of CABA in solving the 3D compressible viscous flow.

**Table 3:** Comparison of the present results against reference for supersonic flow around a sphere

	*θ*	*L*	*L*_*s*_
Ref [38]	150.0	0.36	0.20
Ref [44]	150.9	0.38	-
CABA	150.6	0.40	0.20

### Laminar flow around a delta wing

In the last example, the laminar delta wing is considered to test the improved multi-valued ghost-cell method in parallel framework. The leading and trailing edges of the delta wing are both sloped and sharp, which means a large number of multi-value points will be generated. And this makes delta wing one of the most difficult shapes to handle with GCM. A delta wing model with 75 degrees of sweep is chosen, and it was used in the test of SU2 [45]. The inflow Mach number equals to 0.3 at an angle of attack *α*=12.5^∘^, and Reynold number *Re*=4000, which is similar to Ref. [41]. In this condition, separated zones will be generated at a large angle of attack. Therefore, we select this example to verify the ability of CABA to capture three dimensional dynamic separation vortices, as well as the effectiveness of the improved multi-valued ghost-cell method in large-scale parallelism. The case was tested on a cluster using three nodes (384 cores, two Intel Gold 6149 CPUs at 3.1 GHz per node).

**Fig. 29:**
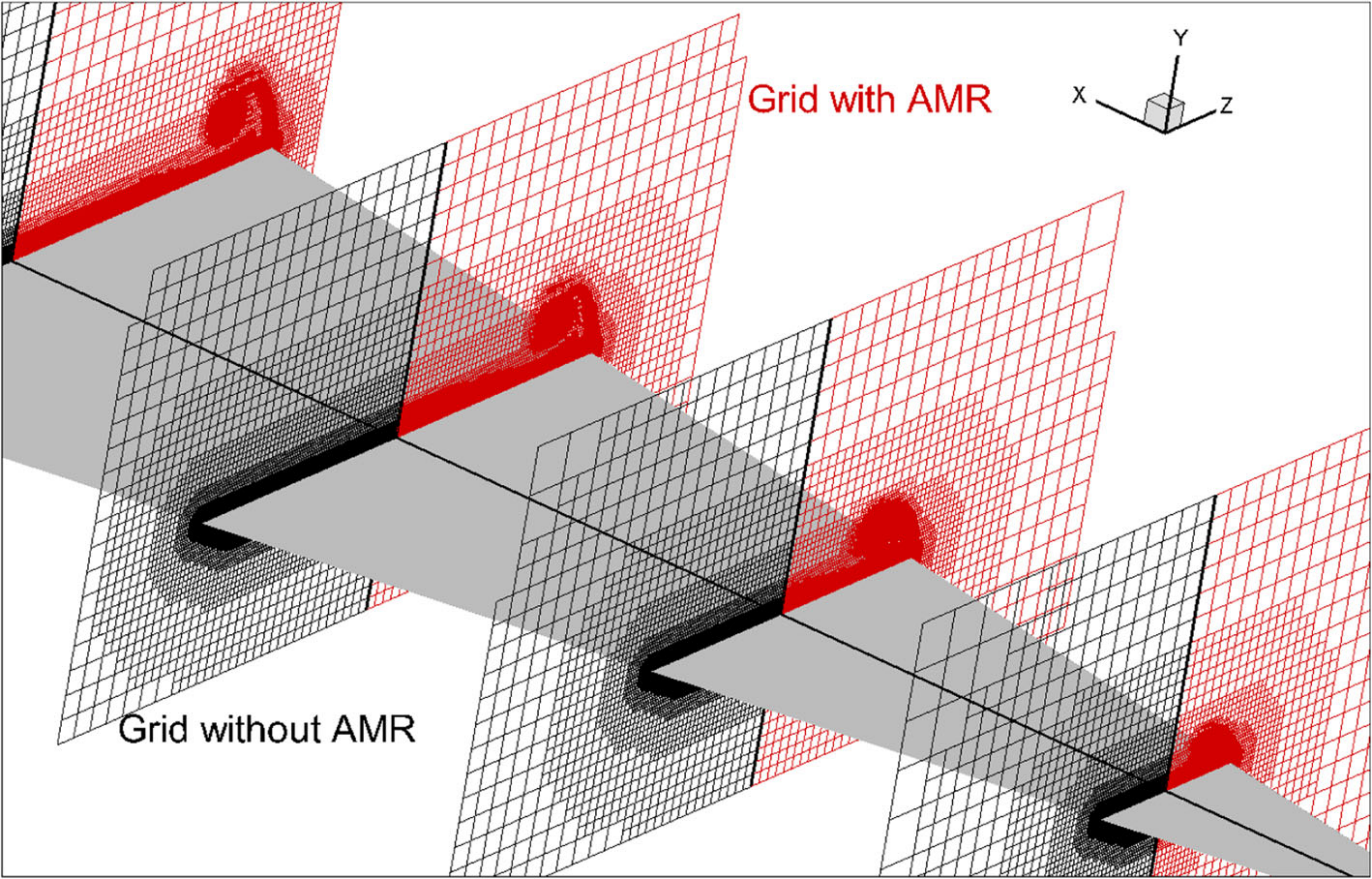
Comparison of the grid with and without AMR

**Fig. 30:**
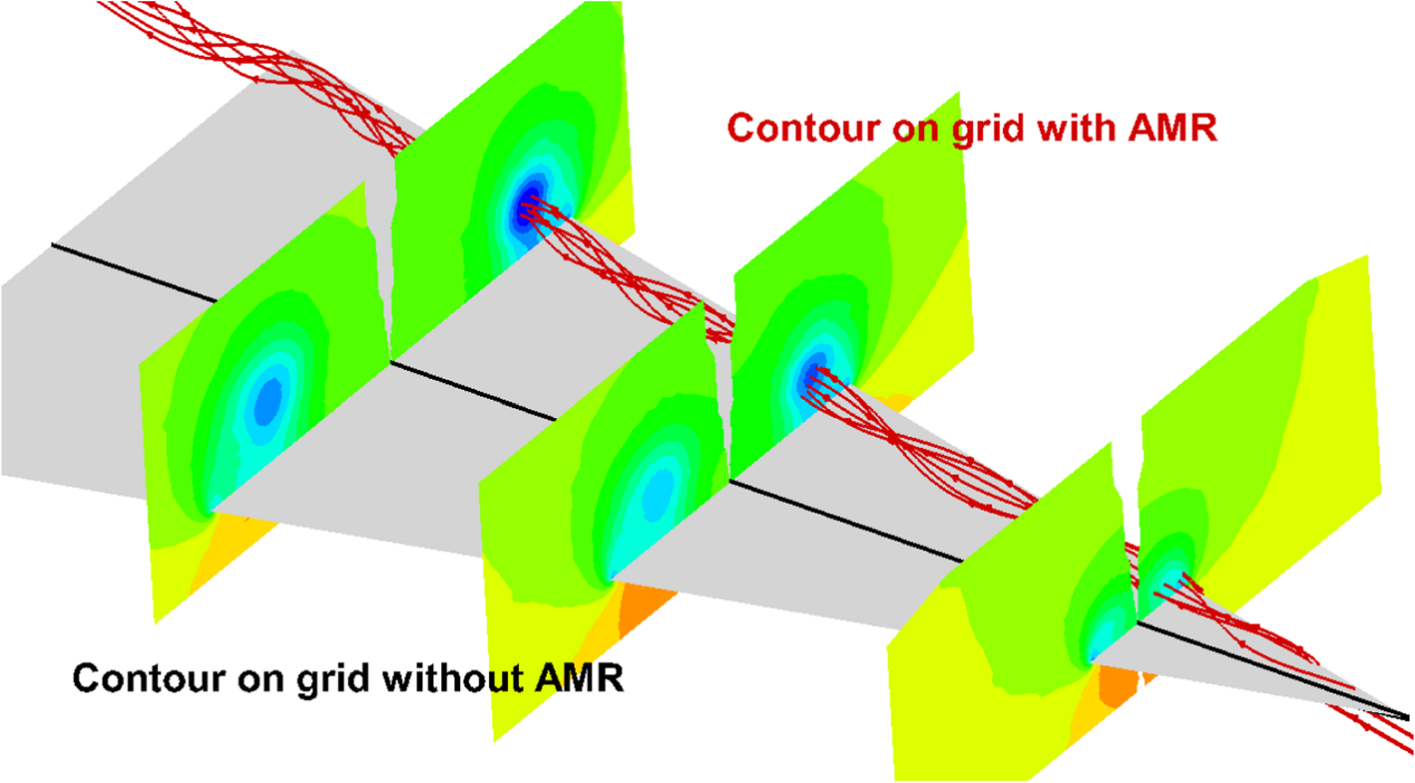
Streamline and pressure contours on grids with AMR over the right half of the wing and grids without AMR over the left half

The computational domain is defined as [0,16]×[0,16]×[0,16], and the initial grid step size is set to 1. The number of the grid is 36,954,569 and increased to over 60 million after solution-based AMR. The comparisons of the grid with and without solution-based AMR on different slices are shown in Fig. 29, from which it can be seen that the grids in the vortex region are fully refined. Figure 30 shows the streamlines and pressure contours resulting from grids with and without AMR. Results obtained on grids without AMR are shown over the left half of the wing while the results from grids with AMR are over the right half. The streamlines illustrate that as the flow passes the leading edge it rolls up and creates a vortex. By comparing the contours on the left and right sides, it can be clearly seen that the vortex calculated on the adaptive grid can be better maintained. The parallel simulation of a three-dimensional thin object such as a delta wing can validate the method in this article, and is of great significance to the automation and robustness of Cartesian grids method.

## Conclusion

The methodology for automatic and efficient simulation of compressible flow on parallel adaptive Cartesian grid is developed in this work. To be specific, with the improved axis aligned bounding box theorem, the grid generator can generate tens of millions of grids for complex three-dimensional bodies in ten minutes automatically and parallelly. Besides, an information transmission approach for the wall boundary is proposed to guarantee the parallelized implementation of the ghost-cell method. Especially, a multi-valued ghost-cell method for handling thin objects is developed to adapt to the parallel framework. Through the combination of these mentioned essential approaches and the open-source library p4est (a Cartesian-based AMR parallel library), a parallel compressible flow solver named CABA is developed.

Then, the overall performance of this numerical method is validated by several inviscid/viscous flow cases, including flow over multiple objects, separated flow, steady and unsteady flow with shock and vortex, as well as the flow around a thin delta wing. The obtained results indicate the capability and parallel scalability of the present methodology are very agreeable as compared with related reference data. As future work, the simulation of high Reynolds number compressible flow is on the agenda.

## Data Availability

The datasets used and/or analyzed during the current study are available from the corresponding author on reasonable request.
